# Xenogeneic Mitochondrial Transplantation Improves Selected Age‐Associated Phenotypes in Mice

**DOI:** 10.1002/advs.75806

**Published:** 2026-05-22

**Authors:** Wenpeng Li, Xijia Wang, Yaning Hu, Zhen Yang, Tian Niu, Xingbo Zhao

**Affiliations:** ^1^ State Key Laboratory of Animal Biotech Breeding College of Animal Science and Technology China Agricultural University Beijing China

**Keywords:** ageing, age‐related features, mitochondrial heteroplasmy, mitochondrial quality control, xenogeneic mitochondrial transplantation

## Abstract

Xenogeneic mitochondrial transplantation (xeno‐MT) improves selected age‐associated phenotypes and mitochondrial functional readouts in mice while engaging host mitochondrial quality‐control‐related pathways. Donor mitochondrial preparations with impaired membrane potential retained measurable activity, but both respiratory competence and in vivo efficacy declined progressively with more extensive room‐temperature damage and were largely lost after complete disruption. Although beneficial effects were observed in additional donor contexts, the present study provides the most detailed in vivo evidence for yak‐derived xeno‐MT, and broader donor equivalence remains to be established. These findings support xeno‐MT as proof‐of‐concept evidence of biological activity and short‐term tolerability under the conditions tested, while long‐term safety, germline relevance, and pathway‐specific dependence remain to be defined.

## Graphical abstract

Yak‐derived xenogeneic mitochondrial transplantation improves selected age‐associated phenotypes in mice, enhances mitochondrial functional readouts, and engages host mitochondrial quality‐control pathways. Broad tissue biodistribution, increased ATP production and mtDNA copy number, reduced ROS levels and dysfunctional mitochondria, improved motility‐related outcomes, and short‐term tolerability support xeno‐MT as proof‐of‐concept biological activity in aging‐related contexts.

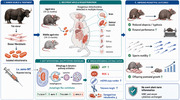

## 1 Introduction

Mitochondrion, present in eukaryotic cells, serves primary functions such as oxidative phosphorylation, the production of reactive oxygen species, and heat generation [1]. Thus, among the key characteristics of aging, mitochondrial DNA (mtDNA) instability is a significant contributor to aging and age‐related pathologies. Strategies aimed at avoiding, mitigating, or correcting mtDNA mutations could potentially help extend both healthspan and lifespan [2].

Mitochondrial transplantation (MT) has shown potential in enhancing mitochondrial respiratory capacity, thereby improving conditions related to disease and aging [3]. In recent years, an increasing number of MT studies have substantiated its therapeutic efficacy. For example, astrocytes regulate the integrity of the blood–brain barrier through MT [4]. Additionally, modified mitochondria, equipped with nanomotors, can even be administered orally and target ischemic areas of the heart, significantly restoring cardiac function in a rat model of ischemic heart disease, with the improvement sustained for up to two weeks after treatment cessation [5]. Transplanted mitochondria isolated from wild‐type mice have been shown to extend lifespan, improve neurological function, and increase energy expenditure in mice with Leigh syndrome [6]. Moreover, several therapeutic applications for administering exogenous mitochondria have been explored [7].

To date, substantial advancements in MT research have utilized a variety of mitochondrial sources, including autologous, allogeneic, and xenogeneic mitochondria [8, 9]. In vitro analyses have demonstrated that when mouse mitochondria are transplanted into human cell lines, host cells exhibit minimal resistance, indicating a high degree of compatibility with exogenous mitochondria [10]. While studies have shown that xenogeneic MT (xeno‐MT) enhances respiratory function in host cells [11, 12, 13, 14], there is evidence suggesting that, in the long term, xenogeneic transplants are less efficient than allogeneic transplants [15]. However, given the limited availability and ethical concerns surrounding allogeneic mitochondria, xenogeneic mitochondria may provide a practical alternative donor source in some settings. Nevertheless, the extent to which therapeutic effects are generalizable across donor sources and their long‐term safety and broader translational applicability remains to be more fully defined.

The mechanisms of MT between cells have been well‐studied, but the effects of using xeno‐MT to delay aging in middle‐aged and elderly mice are not yet fully understood. In this study, we demonstrate that xeno‐MT improves several selected age‐associated phenotypes, including physical performance in aged mice, sperm motility in middle‐aged male mice, and offspring growth‐related outcomes in middle‐aged female mice. Moreover, the preservation of mitochondria post‐isolation remains an important issue in MT research. Our findings suggest that xeno‐MT may have relevance to selected age‐associated phenotypes and mitochondrial dysfunction, while questions regarding long‐term safety, immune consequences, and translational applicability require dedicated future investigation.

## 2 Results

### 2.1 Phenotypic Variations between Xeno‐MT‐Treated and Control Mice

**FIGURE 1:**
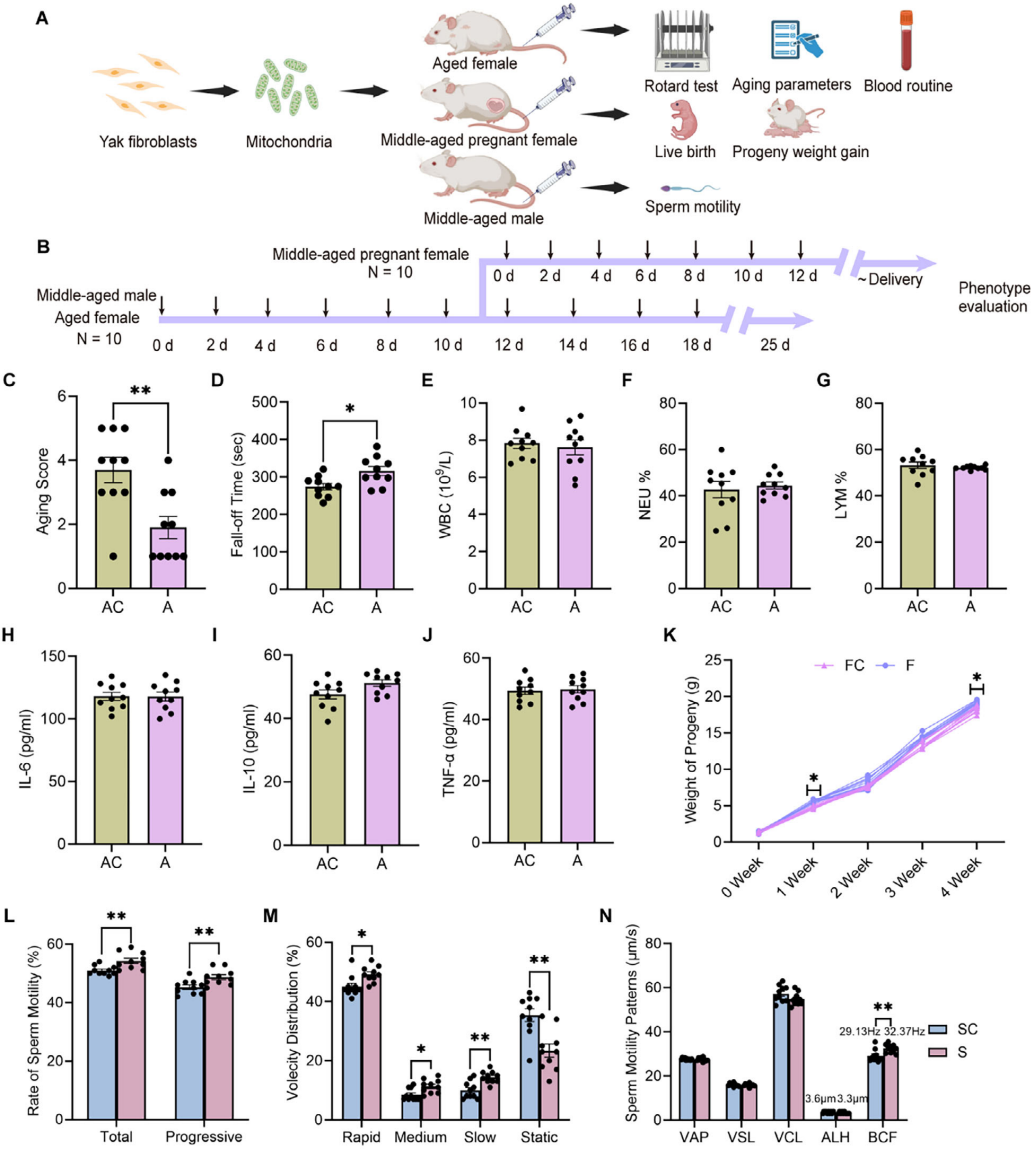
Xenogeneic Mitochondrial Transplantation Improves Phenotypic and Functional Parameters in Aged and Middle‐Aged Mice. (A) Schematic of the experimental design for in vivo xeno‐MT and subsequent phenotypic evaluations. AC, aged control mice; A, xeno‐MT‐treated aged mice. (B) Timeline of the animal treatment protocol across all experimental groups. Arrows indicate the timing of each mitochondrial transplantation (or vehicle control) administration across the treatment schedule. (C) Scoring of conspicuous age‐related phenotypes (including alopecia and kyphosis) in 18‐month‐old mice. Phenotypes were scored from 0 (none) to 3 (high). n = 10 mice per group. (D) Rotarod endurance performance of aged mice. Data represent the latency to fall. n = 10 mice per group. (E–G) Complete blood count analysis showing (E) white blood cell count, (F) neutrophil percentage, and (G) lymphocyte percentage. n = 10 mice per group. (H–J) Serum cytokine levels measured by ELISA: (H) IL‐6, (I) IL‐10, and (J) TNF‐α. n = 10 mice per group. (K) Weekly weight gain of offspring from middle‐aged female mice. FC, middle‐aged female control mice; F, xeno‐MT‐treated middle aged female mice. n = 10 litters per group. (L) Sperm motility analysis in middle‐aged male mice. SC, middle‐aged male control mice; S, xeno‐MT‐treated middle‐aged male mice. n = 10 mice per group. (M) Distribution of spermatozoa by velocity category. n = 10 mice per group. (N) Average velocity of motile sperm. n = 10 mice per group. Statistical significance for the ordinal phenotype scores in panel C was assessed using the Mann–Whitney U test. For the longitudinal offspring body‐weight data in panel K, statistical significance was assessed using a mixed‐effects model with treatment group and time as fixed effects. For the remaining two‐group comparisons, statistical significance was assessed using two‐tailed unpaired Student's t test when normality assumptions were met; otherwise, the Mann–Whitney *U* test was used. Data are expressed as mean ± SEM. ^*^ *p* < 0.05, ^**^ *p* < 0.01.

Unless otherwise indicated, phenotypic and tissue‐level assessments were performed after completion of the 10‐dose treatment schedule shown in Figure 1A, B. The xeno‐MT group exhibited fewer signs of aging (Figure 1C, Figure S1A), particularly in alopecia and kyphosis (Table S1). In the rotarod test, aged mice that underwent xeno‐MT (A group) exhibited a significantly longer duration of 316.40 ± 12.09 s on the rod compared to aged control mice (AC) who remained on the rod for only 274.60 ± 8.65 s (Figure 1D). Moreover, xeno‐MT treatment did not cause significant changes in white blood cell count (Figure 1E), neutrophil percentage (Figure 1F), or lymphocyte percentage (Figure 1G). The ELISA results showed that the levels of cytokines IL‐6, IL‐10, and TNF‐α in the serum of the A and AC groups were comparable (Figure 1H–J). These results indicate that, under the conditions tested in the present study, xeno‐MT did not produce overt short‐term abnormalities in basic hematologic indices or the limited serum cytokines examined.

Pups born to middle‐aged female mice that underwent xeno‐MT (F group) during pregnancy gained more weight every 7 days than pups born to the control group (FC group) (Figure 1K). There were no significant differences between groups in the number of live‐born pups per litter (Figure S1B). Exogenous mitochondria were also detected in the offspring (Figure S1C,D). Moreover, middle‐aged male mice that received xeno‐MT (S group) produced more active sperm, with significantly higher total motility compared to the control group (SC group) (Figure 1L). The velocity distribution of motile sperm in xeno‐MT male mice showed a significant increase in rapid, medium, and slow‐speed sperm, resulting in a significant reduction in static sperm compared to the control group (Figure 1M). Additionally, the beat frequency of sperm in xeno‐MT male mice was higher than that in the control group (Figure 1N).

### 2.2 Observations of Exogenous Mitochondria in Xeno‐MT Mice

**FIGURE 2:**
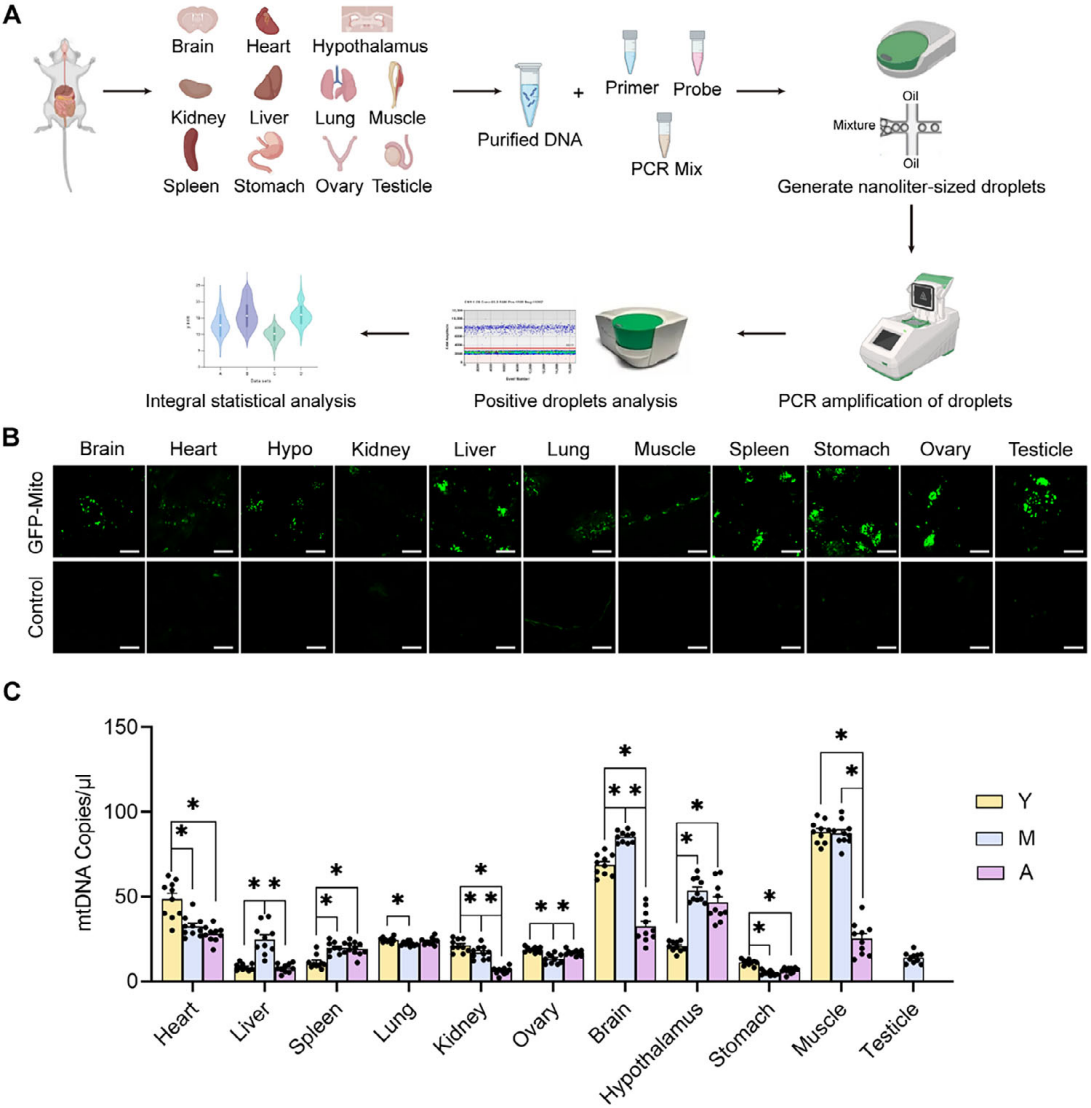
Biodistribution and Quantification of Exogenous Mitochondria Following; Xenogeneic Transplantation. (A) Schematic representation of the droplet digital PCR (ddPCR) workflow for the absolute quantification of exogenous mitochondrial DNA (mtDNA). The process illustrates droplet generation, endpoint PCR amplification, and fluorescence‐based detection of target sequences. (B) Representative confocal microscopy images showing the presence of exogenous mitochondria (labeled with GFP) in various host tissues, including the hypothalamus (Hypo). Images confirm the uptake and retention of transplanted organelles. (C) Absolute quantification of exogenous (yak‐derived) mtDNA copy numbers per microliter of genomic DNA extracted from indicated tissues of young (Y), middle‐aged (M), and aged (A) mice. Data are derived from ddPCR analysis using species‐specific primers and probe. n = 10 mice per group. Scale bars = 20 µm. For panel C, statistical significance was assessed using one‐way ANOVA followed by Tukey's multiple comparisons test. For tissue‐wise comparisons within this endpoint family, *p* values were additionally adjusted using the Benjamini–Hochberg procedure. Data are expressed as mean ± SEM. ^*^ *p* < 0.05.

A total of 11 different types of organs and tissues were collected to assess the persistence of exogenous mitochondria (Figure 2A). We observed extensive evidence of exogenous mitochondria in all tissues of xeno‐MT treated mice (Figure 2B). Notably, the highest levels were found in brain and skeletal muscle of both xeno‐MT‐treated young (Y group) and xeno‐MT treated middle‐aged (M group) mice (Figure 2C, Figure S2).

Mice in A group showed significantly lower retention of exogenous mitochondria in the heart, kidney, brain, stomach, and skeletal muscle compared to the Y mice. Additionally, in A mice, the liver, kidney, brain, and skeletal muscle exhibited significantly reduced mitochondrial retention compared to the M mice. Furthermore, the heart, ovary, and stomach of M mice contained significantly lower levels of exogenous mitochondria than those of the Y mice.

### 2.3 Assessment of ATP, ROS, mtDNA Copy Number, mtDNA D‐loop Methylation, and Telomere Length in Treated and Control Mouse Tissues

**FIGURE 3:**
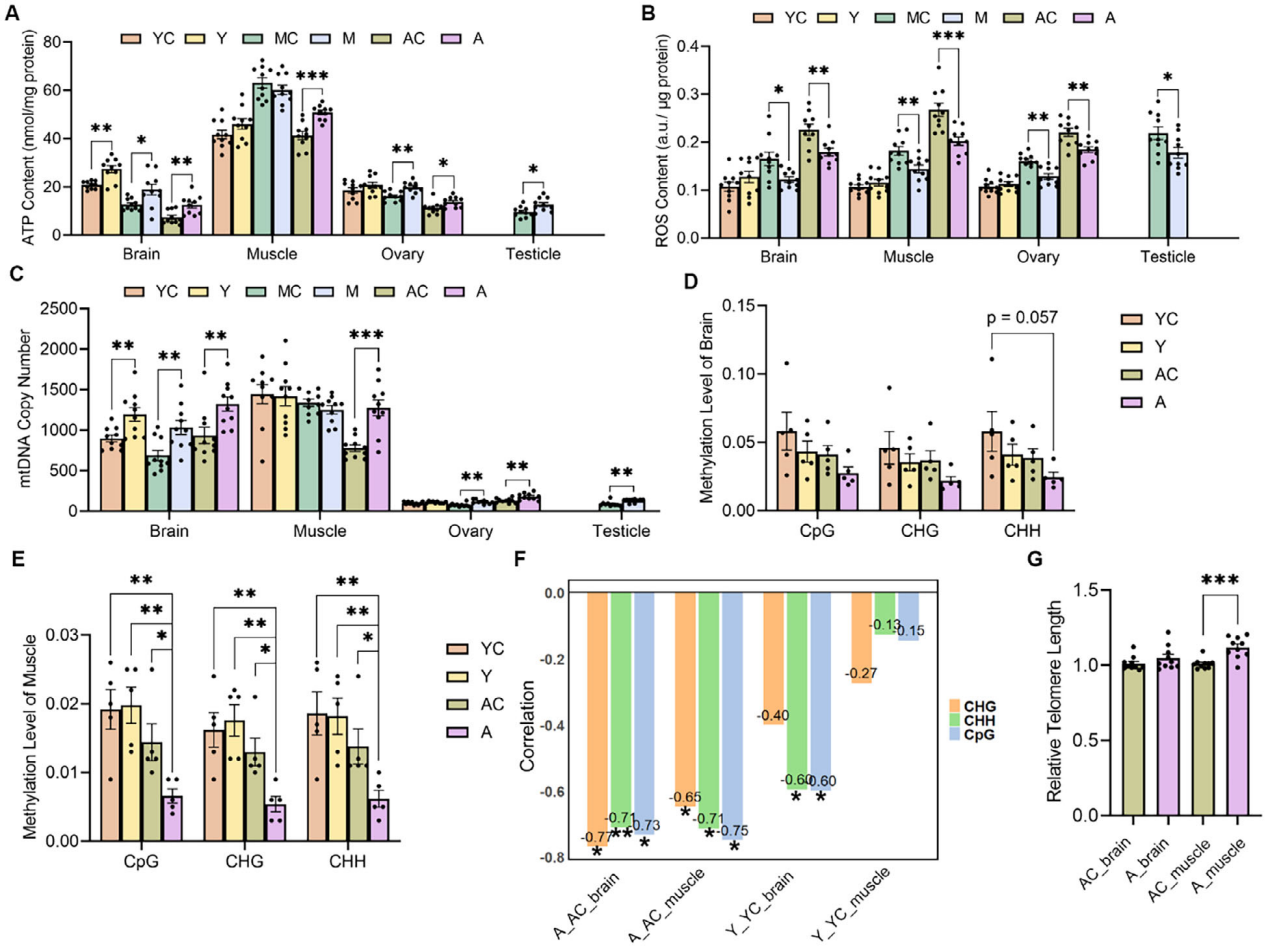
Xeno‐MT Modulates Tissue Bioenergetics, Mitochondrial Genome Replication, and Telomere Length in an Age‐Dependent Manner. (A, B) ATP content (nmol/mg protein) and ROS level (a.u./ µg protein) in different tissues of treated and control mice. n  =  10 mice per group. (C) mtDNA copy numbers detected in different tissues of treated and control mice. n  =  10 mice per group. (D, E) Methylation levels at CpG, CHG, and CHH sites in the mtDNA LSP and OH regions in different tissues of treated and control mice. n  =  5 mice per group. (F) The Spearman correlation between mtDNA copy number and methylation levels. YC/ MC/AC, young/middle‐aged/aged control. Y/M/A, young/middle‐aged/aged mito‐treated group. (G) Relative telomere length in brain and muscle of treated and control mice. n  =  10 mice per group. For panels A–C, statistical significance was assessed within each tissue using two‐way ANOVA with age and treatment as factors, followed by Sidak's multiple comparisons test for pre‐specified within‐age contrasts. For panels D, E and G, statistical significance was assessed using one‐way ANOVA followed by Tukey's multiple comparisons test. For panel F, correlations were assessed using Spearman's rank correlation. *P* values for tissue‐wise treatment comparisons within each endpoint family were additionally adjusted using the Benjamini–Hochberg procedure. Data are expressed as mean ± SEM. ^*^ *p* < 0.05, ^**^ *p* < 0.01, ^***^ *p* < 0.001.

For Y mice, exogenous mitochondria increased ATP levels (nmol/mg protein) in the brain, kidney, and liver, but did not reduce ROS content (a.u./ µg protein) in any of the tested tissues. For M mice, xeno‐MT enhanced ATP levels in the brain, hypothalamus, kidney, liver, lung, testicles, and ovaries. Additionally, ROS levels were reduced in the brain, kidney, muscle, ovary, and testicle. For A mice, energy production in the brain, heart, hypothalamus, liver, muscle, and ovary was restored, and ROS levels were reduced in all organs except the lung and spleen following xeno‐MT treatment (Figure S3A,B, Figure 3A,B). A significant increase in mtDNA copy number was observed in the brain, kidney, liver, ovary, and stomach of Y mice, in the brain, kidney, liver, lung, muscle, ovary, spleen, and testicle of M mice, and in the brain, heart, hypothalamus, kidney, lung, muscle, ovary, and spleen of A mice (Figure S3C, Figure 3C). Across tissues, ATP, ROS, and mtDNA copy number were analyzed within an age‐by‐treatment framework, and tissue‐wise treatment comparisons within each endpoint family were additionally adjusted using the Benjamini–Hochberg procedure.

Given the elevated mtDNA copy numbers observed across various organs in xeno‐MT‐treated mice relative to controls, the role of the LSP and O_H_ regions in mitochondrial replication was scrutinized. Methylation levels at these loci were quantified to investigate the underlying cause of the increased mtDNA copy numbers in xeno‐MT‐treated mice. In the brain, methylation levels at CpG, CHG, and CHH sites in the LSP and O_H_ regions of A mice were lower compared to young control mice (YC) (Figure 3D). In muscle, these methylation levels in A mice were significantly reduced in comparison to all other groups (Figure 3E). Furthermore, a significant negative correlation was observed between the methylation levels in the brain and muscle tissues of aged (A and AC) mice and their respective mtDNA copy numbers (Figure 3F). The relative telomere length in muscle tissue of A mice was significantly longer than that of aged control (AC) mice (Figure 3G).

### 2.4 Variants Detection Through Whole Genome Sequencing

Given the higher copy numbers of exogenous mtDNA observed in the brain and muscle tissues of the Y and M groups, as well as the significant improvements in ATP levels, ROS levels, and mtDNA copy numbers in these tissues in the A group, we selected brain and muscle tissues for mechanistic exploration. Whole‐genome sequencing was performed on the muscle and brain tissues of three groups of mice: A, AC, and YC. Significant SNPs and InDels were identified by comparing the filtered variants between pairs of groups (A‐AC, A YC, and AC‐YC). The number of significant variants between the aged groups (A‐AC) was low (Figure S4A), while the number of variants between the aged and young groups (AC‐YC, A‐YC) was much higher (Figure S4B,C). Gene Ontology (GO) enrichment analysis was conducted on the significant inter‐group variants. Due to the high similarity of genome‐wide variants between the brain and muscle tissues of the same individual and the similar enrichment results of A‐YC and AC‐YC, only the enrichment analysis results for A‐AC and AC‐YC in muscle tissue are presented.

**FIGURE 4:**
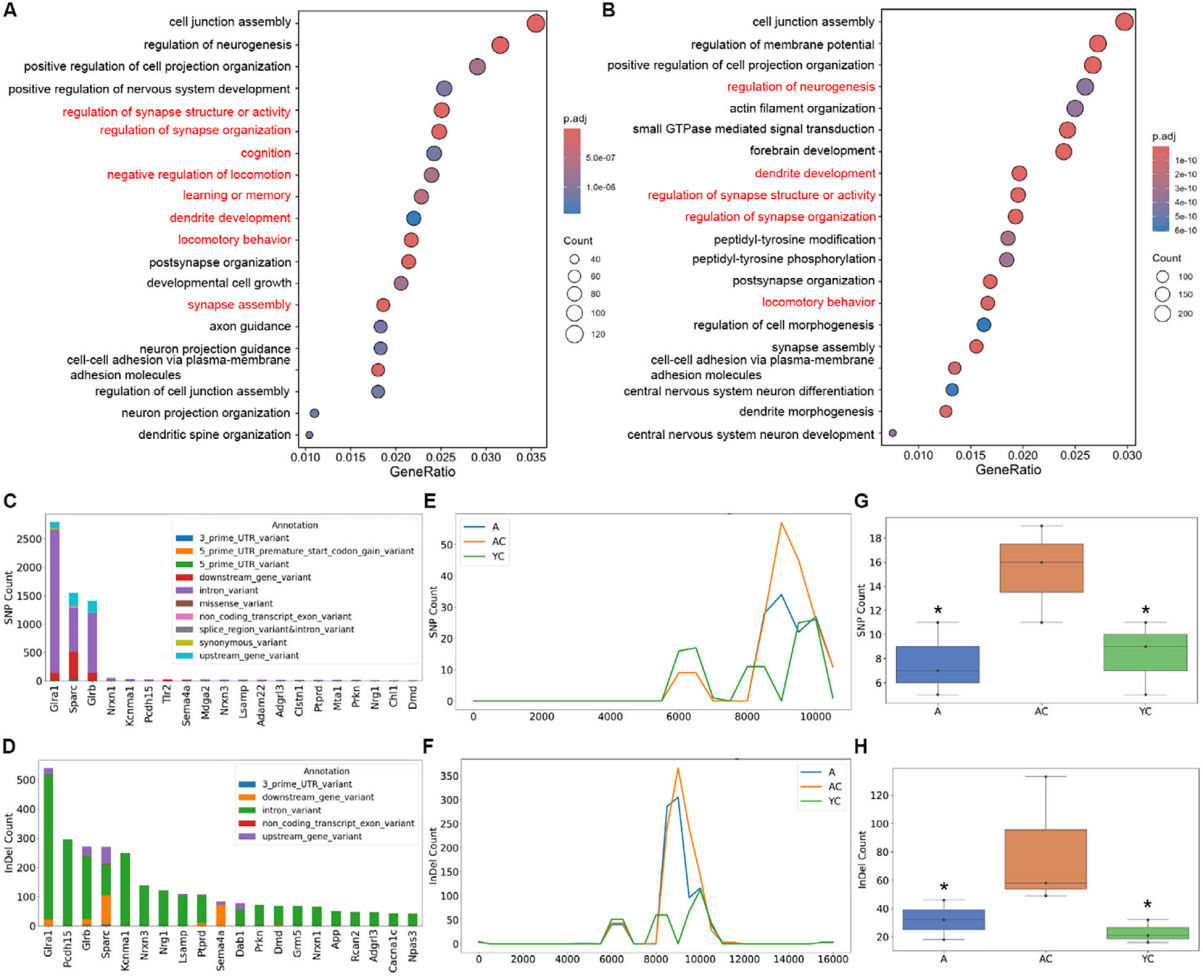
Whole‐Genome Sequencing Reveals Altered Nuclear and Mitochondrial Genetic Landscapes Following Xeno‐MT Treatment. (A) GO enrichment of significant SNPs between A and AC groups. (B) GO enrichment of significant SNPs between AC and YC groups. (C) The top 20 genes with the highest number of significant SNPs (A‐AC) and their distribution. (D) The top 20 genes with the highest number of significant InDels (A‐AC) and their distribution. (E) Sliding window analysis of the brain mitochondrial genome SNPs across A, AC and YC groups. (F) Sliding window analysis of the muscle mitochondrial genome InDels across A, AC and YC groups. (G) SNP count in the 9500–10 500 region of the brain mitochondrial genome. (H) InDel counts in the 9500–10 500 region of the muscle mitochondrial genome. n = 3 mice per group. For genome‐wide SNP and InDel frequency comparisons, statistical significance was assessed using the Chi‐square test of independence, with p values adjusted using the Benjamini–Hochberg false discovery rate method. For panels G and H, statistical significance was assessed using one‐way ANOVA followed by Tukey's multiple comparisons test. Data are expressed as mean ± SD. ^*^ *p* < 0.05.

In the A‐AC enrichment results, significant SNP and InDel enrichment was observed in pathways related to regulation of synapse structure or activity, cognition, learning or memory, and locomotory behavior (Figure 4A), nominating candidate pathways potentially relevant to the age‐associated phenotypes examined here. Similarly, the AC‐YC SNP enrichment results also included related GO terms such as regulation of synapse structure or activity and locomotory behavior (Figure 4B). The GO terms enriched in the InDel results are shown in Figure S4D,E. Among the genes enriched in the aforementioned GO terms, the top 20 genes with the most SNPs and InDels were selected (Figure 4C,D), and most of the SNPs and InDels were located in the intron region. Among the genes recurrently identified in these analyses, *Prkn* emerged as a candidate gene of interest, rather than as direct mechanistic proof. Mitochondrial genome variations were analyzed using a sliding window approach (window size 1000 bp, step size 500 bp), revealing the highest inter‐group differences in the 9500–10 500 bp region (Figure 4E,F, Figure S5). Among these, the number of variations was lowest in the YC group and highest in the AC group. In the brain, the number of SNPs in the A group was significantly lower than in the AC group (Figure 4G), while in muscle, the number of InDels in the A group was significantly lower than in the AC group (Figure 4H).

### 2.5 Gene Expression Profiles Analysis Using RNA‐seq and WGBS

**FIGURE 5:**
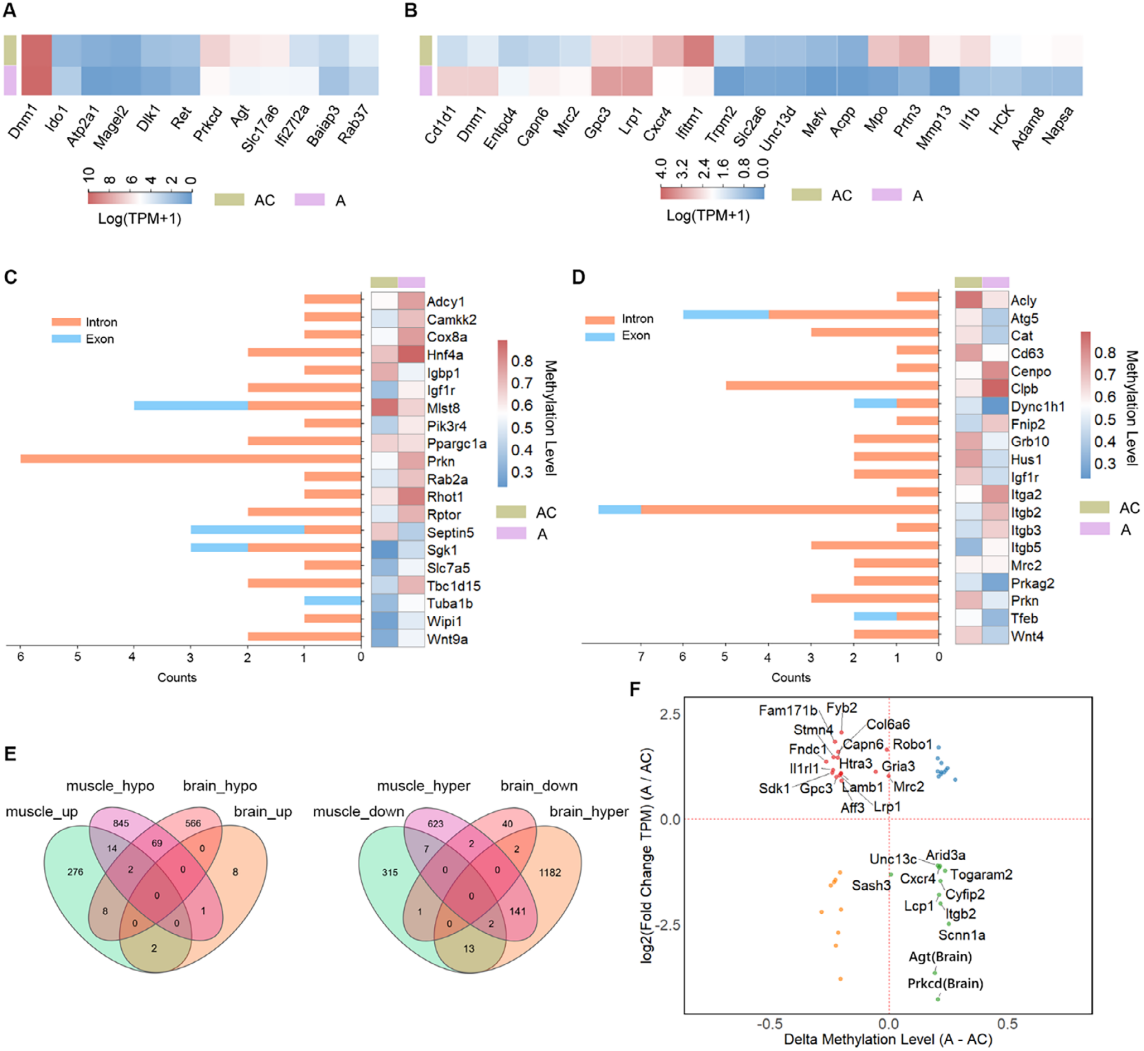
Integrated Multi‐Omics Analysis Reveals Epigenetic and Transcriptional Reprogramming Linked to Autophagy and Mitochondrial Function Following Xeno‐MT Treatment. (A) Expression of Dnm1 and autophagy‐related genes in brain. (B) Expression of Dnm1 and autophagy‐related genes in muscle. (C) Methylation levels and intron/exon counts in DMRs of aging‐process‐related gene in brain, each gene's occurrence at the same intron or exon position is counted only once. (D) Methylation levels and intron/exon counts in DMRs of aging‐process‐related gene in muscle. (E) The intersection of upregulated/downregulated and hypermethylated/hypomethylated genes in the two tissues. (F) Gene symbols of hypomethylated and upregulated genes, and hypermethylated and downregulated genes in muscle and brain. Statistical significance was calculated using a Poisson‐based model in DEGseq, where *p*‐values were determined for differences in gene expression between groups. n = 3 mice per group. For RNA‐seq analyses, differential expression was assessed using DEGseq, with *p* values adjusted using the Benjamini–Hochberg and Storey–Tibshirani methods; genes with fold change > 2 and *q* value ≤ 0.001 were considered significant. The differences of expression levels of a few candidate genes highlighted in panels A–B is not obvious from the heatmap provided, adjusted statistics are provided in Tables S4 and S5. Data shown in panels A–D and F should be interpreted as candidate transcriptomic and methylation‐associated signals rather than direct mechanistic proof.

We then performed transcriptome and whole‐genome methylation analyses to identify treatment‐responsive molecular changes in aged tissues after xeno‐MT and to nominate candidate pathways and genes for subsequent mechanistic investigation. Because these experiments were designed as aged‐treated vs. aged‐control comparisons, they were intended for candidate discovery within the aged background rather than for determining whether xeno‐MT restores a global youthful transcriptomic or epigenetic state. To investigate the effect of xeno‐MT treatment on gene expression, RNA‐seq was performed on the muscle and brain tissues of groups A and AC. In the brain, 11 genes were significantly upregulated, and 45 genes were significantly downregulated. In muscle, 302 genes were significantly upregulated, and 338 genes were significantly downregulated (Figure S6A). Notably, the top 20 KEGG pathways enriched for significantly differentially expressed genes (DEGs) in muscle included the Phagosome pathway (Figure S6B). In the brain, the top 20 KEGG pathways included Development and regeneration, Signaling molecules and interaction, Signal transduction, and Endocrine system pathways, which are potentially related to aging regulation (Figure S6C). The expression of *Dnm1*, a gene encoding a protein homologous to *Drp1*, which is essential for maintaining mitochondrial network dynamics, was differentially expressed in both the brain and muscle. (Figure 5A,B).

To explore the impact of xeno‐MT treatment on genome‐wide methylation, WGBS sequencing was performed on the muscle and brain tissues of groups A and AC. In the brain, there were 2228 hypermethylated differentially methylated regions (DMRs) and 1089 hypomethylated DMRs. In muscle, there were 1502 hypermethylated DMRs and 1659 hypomethylated DMRs (Figure S6D). Enrichment analysis of genes located in these DMRs showed significant methylation differences in autophagy‐related genes in both the brain and muscle, with enriched pathways including the mTOR signaling pathway, AMPK signaling pathway, mitophagy, autophagy, and longevity regulating pathway (Figure S6E,F). After xeno‐MT treatment, the methylation levels of certain autophagy and aging related genes in the brain and muscle were significantly altered (Figure 5C,D). Most of the DMRs were located in the intron regions of the selected genes. Among these, the *Prkn* gene exhibited significant methylation level differences in both muscle and brain. To further analyze the deeper impact of xeno‐MT treatment on gene expression, a combined RNA‐seq and WGBS analysis was performed. Intersection analysis of DEGs and significantly differentially methylated genes (DMGs) revealed that there were 16 genes that were both hypomethylated and upregulated, and 9 genes that were both hypermethylated and downregulated in muscle. In the brain, only 2 genes were both significantly hypermethylated and downregulated (Figure 5E,F). Among these intersecting genes, *Prkn*, *Mrc2*, *Itgb2*, *Lrp1*, *Capn6*, and *Prkcd* were best viewed as candidates for further mechanistic validation.

Overall, these transcriptomic and methylation‐associated changes nominate treatment‐responsive pathways and candidate genes for future validation, but they should not be interpreted as direct proof that xeno‐MT restores a specific mitochondrial quality‐control pathway in vivo.

### 2.6 Chloroquine‐Sensitive Autophagic Flux and Mitochondrial Quality‐control Remodeling Are Associated With the Mitochondrial Benefits of Xeno‐MT

**FIGURE 6:**
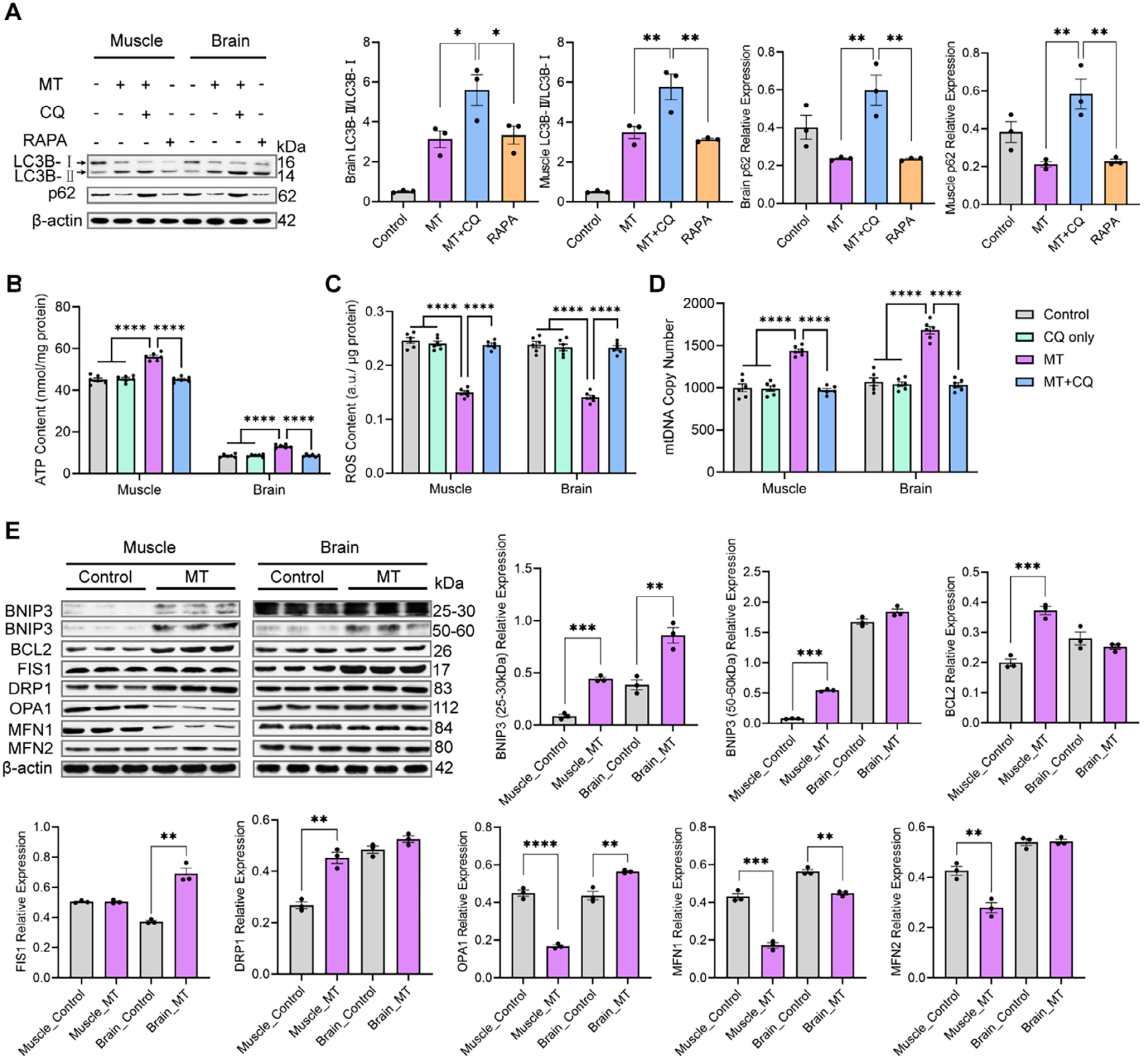
Xeno‐MT Enhances Chloroquine‐sensitive Autophagic Flux and This Response Contributes to Mitochondrial Functional Improvement in Aged Mice. (A) Analysis of autophagic flux in brain and muscle using LC3B and p62. CQ was used as a lysosomal inhibitor to block autophagosome degradation, and RAPA as a positive control for autophagy induction. n = 3 mice per group. (B) ATP content in the indicated groups. n = 6 mice per group. (C) ROS levels in the indicated groups. n = 6 mice per group. (D) mtDNA copy number in the indicated groups. n = 6 mice per group. (E) Expression levels of BNIP3 and BCL2, shown here as stress‐response/survival‐associated proteins relevant to mitochondrial quality‐control interpretation, together with mitochondrial dynamics proteins FIS1, DRP1, OPA1, MFN1, and MFN2. n = 3 mice per group. For panel A, statistical significance was assessed using the Kruskal‐Wallis test followed by Dunn's multiple comparisons test. For panels B–D, statistical significance was assessed using one‐way ANOVA followed by Tukey's multiple comparisons test. For panel E, pairwise comparisons were assessed using the Mann–Whitney *U* test. Data are expressed as mean ± SEM. ^*^ *p* < 0.05, ^**^ *p* < 0.01, ^***^ *p*< 0.001, ^****^ *p* < 0.0001.

To determine whether xeno‐MT was associated with active autophagic flux rather than simple autophagosome accumulation, we performed an autophagic flux assay in aged mice using chloroquine (CQ) as a lysosomal inhibitor to block autophagosome degradation and rapamycin (RAPA) as a positive control for autophagy induction. In this assay, aged mice received either no treatment, xeno‐MT alone, xeno‐MT followed by CQ administration 2 h later, or RAPA alone. After a total of five treatment cycles, tissues were collected 4 h after the final intervention for protein analysis. LC3B protein aggregation was observed in both brain and muscle (Figure S7A–C). Western blot analysis showed that the LC3B‐II/LC3B‐I ratio increased in the xeno‐MT and RAPA groups and increased further after CQ treatment, whereas p62 showed the opposite trend (Figure 6A). Together, these findings support the interpretation that xeno‐MT enhances ongoing autophagic flux in aged tissues, rather than merely causing autophagosome accumulation.

To assess whether this autophagic response was functionally related to the mitochondrial benefits of xeno‐MT, we further compared ATP content, ROS levels, and mtDNA copy number among the control, CQ‐only, xeno‐MT, and xeno‐MT+CQ groups after 5 times treatments. As expected, xeno‐MT significantly increased ATP production, reduced ROS accumulation, and elevated mtDNA copy number relative to the control group. Importantly, these beneficial effects were attenuated by CQ treatment, and the xeno‐MT+CQ group became comparable to the control group for these readouts, whereas the CQ‐only group did not reproduce the beneficial profile observed in the xeno‐MT group (Figure 6B–D). These findings indicate that lysosome‐dependent autophagic flux contributes functionally to the mitochondrial improvements induced by xeno‐MT.

In parallel, we examined several proteins associated with mitochondrial stress responses and mitochondrial network remodeling. Xeno‐MT was associated with increased BNIP3 in both brain and muscle, increased BCL2 in muscle, higher FIS1 in brain, higher DRP1 in muscle, reduced MFN1 in both tissues, and reduced MFN2 in muscle. OPA1 showed tissue‐specific changes, increasing in brain but decreasing in muscle (Figure 6E). Taken together, these molecular changes do not establish pathway‐specific dependence, but they are consistent with broad remodeling of mitochondrial quality‐control‐related pathways and a shift toward fission‐associated mitochondrial maintenance in aged tissues after xeno‐MT.

### 2.7 Establishment of a Fusion‐Based Xenogeneic Mitochondrial Heteroplasmic Cell Model

#### 2.7.1 Generation of YM Cells by Fusion of Denucleated Yak Fibroblasts With TM4 Cells

**FIGURE 7:**
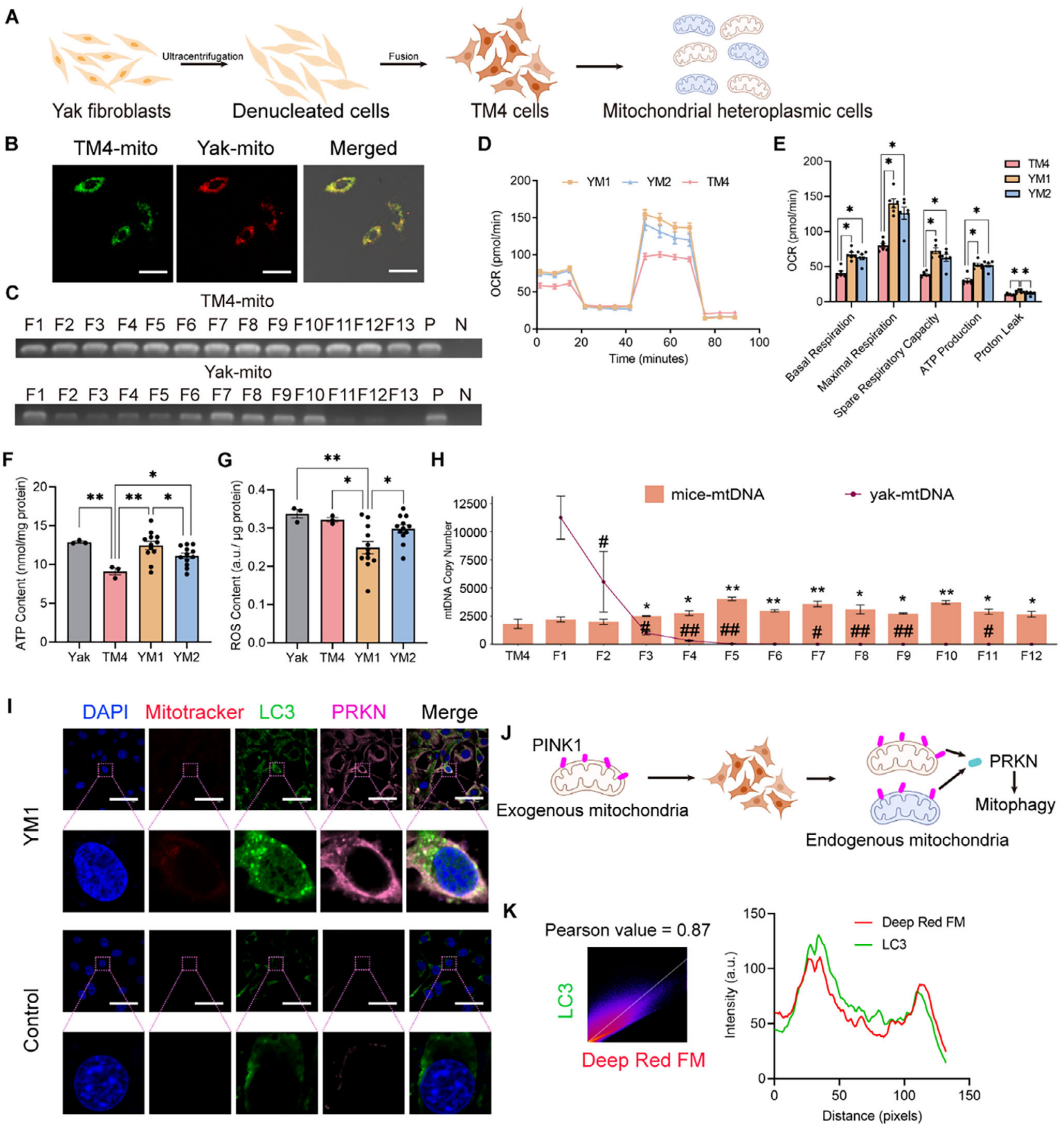
Generation and Characterization of a Fusion‐Based Xenogeneic Mitochondrial Heteroplasmic Cell Model. (A) Schematic diagram of preparation of mitochondrial heteroplasmic cells. (B) Confocal microscopy image showing observation of yak and mouse mitochondria labelled by MitoTracker. (C) Mice and yak mtDNA detection by specific PCR amplification. *P* represents positive control; *N* represents negative control. (D) Oxygen consumption rate (OCR) of heteroplasmic cells and TM4 cells. N = 6 independent experiments. (E) Parameters of mitochondrial respiration, including basal respiration, maximal respiration, spare respiratory capacity, ATP production and proton leak. (F, G) Evaluation of ATP and ROS content of mitochondrial heteroplasmic cells, yak cells and TM4 cells. N = 12 independent experiments. (H) Relative mtDNA copy number of yak and mouse mtDNA. N = 3 independent experiments. (I) Immunofluorescence detection of LC3B+ autophagosomes and PRKN in YM1 cells. (J) Schematic of a candidate PINK1‐Parkin‐associated mitophagy framework relevant to intracellular xenogeneic mitochondrial entry. (K) Co‐localization analysis of LC3B and exogenous mitochondrial fluorescence in YM cells generated by cell fusion. For panels D–G, statistical significance was assessed using one‐way ANOVA followed by Tukey's multiple comparisons test. For panel H, statistical significance was assessed using the Kruskal–Wallis test, followed by Dunn's test for pre‐specified adjacent‐generation comparisons. Data are expressed as mean ± SEM. ^*^ *p* < 0.05, ^**^ *p* < 0.01, ^#^ *p* < 0.05, ^##^ *p* < 0.01. Scale bars = 10 µm.

Because the in vitro fusion model introduces donor mitochondria directly into the recipient cytoplasmic context, whereas the in vivo transplantation model requires extracellular delivery and cellular uptake of free mitochondria, these two systems should not be interpreted as mechanistically identical. To establish a controlled intracellular xenogeneic mitochondrial‐entry model complementary to, but mechanistically distinct from, the in vivo free‐mitochondria transplantation experiments, we generated YM cells by fusion of mouse TM4 cells with denucleated yak fibroblasts (Figure 7A). TM4 cells were used as a reproducible murine recipient background, whereas yak fibroblasts were used to maintain donor‐species consistency with the in vivo xeno‐MT experiments. Several clones exhibiting both red and green fluorescence were identified using a confocal microscope (Figure 7B), indicating successful fusion of TM4 cells with yak mitochondria. A total of 12 clones of mitochondrial heteroplasmic cells were selected and cultured for further investigations. PCR amplification using specific primers successfully detected mtDNA from both yak and mice (Figure 7C). Additionally, the PCR amplification results showed that the exogenous yak mtDNA declined to a level that could no longer be specifically amplified after 4 weeks (within 12 cell generations).

#### 2.7.2 Performance of Mitochondrial Heteroplasmic Cells

We monitored mitochondrial respiration in live cells using extracellular flux analyzer. The mitochondrial respiration of the fourth generation of YM cells (YM1) and the 11th generation of YM cells (YM2) outperformed TM4 cells in all aspects (Figure 7D,E). To independently verify energy production and ROS content using a commercial kit, YM1 cells exhibited significantly higher ATP levels compared to both YM2 cells and TM4 cells (Figure 7F). Furthermore, ATP content of YM2 cells was also significantly higher than that of TM4 cells. On the other hand, ROS content in YM1 cells was significantly reduced compared to Yak, TM4, and YM2 cells (Figure 7G). From the third (F3) generation of YM cells, the mouse mtDNA relative copy number was significantly higher than that in TM4 cells (Figure 7H). Except for the sixth (F6), 10th (F10), and 12th (F12) generations of YM cells, the yak mtDNA copy number in each generation of YM cells decreased than its last generation. Immunofluorescence analysis showed co‐localization patterns consistent with activation of mitophagy and involvement of PINK1‐Parkin‐related mitochondrial quality‐control signaling (Figure 7I,J). Colocalization analysis of exogenous mitochondrial fluorescence with LC3 in the fusion‐based heteroplasmic cell model was consistent with activation of intracellular mitophagy‐related responses (Figure 7K).

### 2.8 Functional Comparisons across Donor Sources and Delivery Methods

**FIGURE 8:**
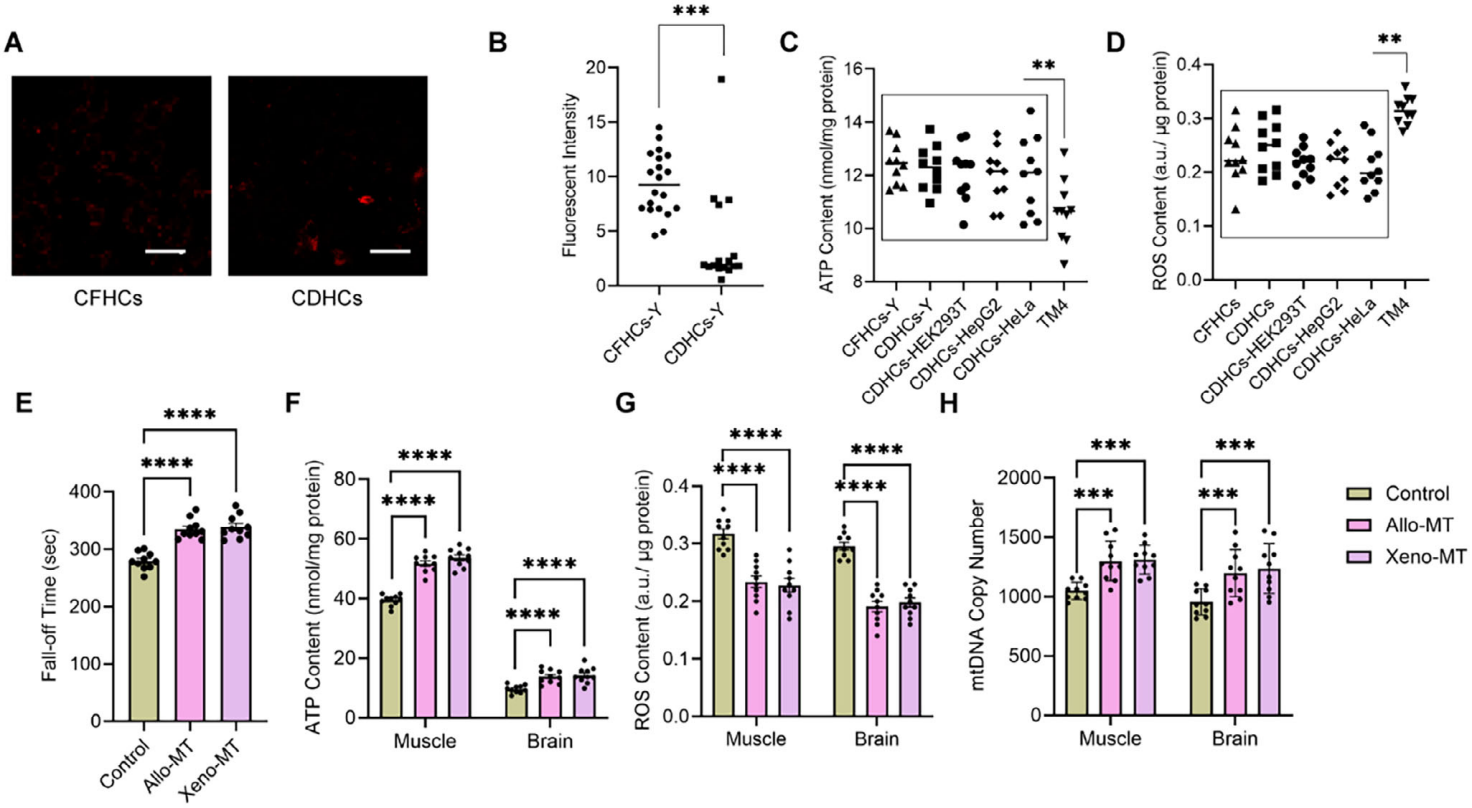
Functional Effects of Mitochondrial Transplantation across Sources and Methods. (A) Mitotracker‐labeled exogenous mitochondria in heteroplasmic cells obtained through cell fusion and co‐culture methods. (B) Quantification of exogenous mitochondria in heteroplasmic cells derived using different methods. N = 10 independent experiments. (C) ATP levels in heteroplasmic cells. N = 10 independent experiments. (D) ROS levels in heteroplasmic cells. N = 10 independent experiments. (E) Latency to fall on the rotarod for aged control (AC), allogeneic mitochondrial transplantation (Allo‐MT), and xenogeneic mitochondrial transplantation (Xeno‐MT) mice. n = 10 mice per group. (F) ATP content in brain and muscle of AC, Allo‐MT, and Xeno‐MT mice. n  =  10. (G) ROS levels in brain and muscle of AC, Allo‐MT, and Xeno‐MT mice. n = 10 mice per group. (H) mtDNA copy number in brain and muscle of AC, Allo‐MT, and Xeno‐MT mice. n = 10 mice per group. CFHCs‐Y, cell‐fusion‐derived heteroplasmic cells with yak mitochondria; CDHCs‐Y, co‐culture‐derived heteroplasmic cells with yak mitochondria. For CDHC method, isolated mitochondria were incubated with recipient cells for 12 h and applied at 100 µg/mL. Data are expressed as mean ± SEM. Statistical significance was assessed by one‐way ANOVA followed by Tukey's multiple comparisons test, ^*^ *p* < 0.05, ^**^ *p* < 0.01, ^***^ *p* < 0.001, ^****^ *p* < 0.0001. Scale bars = 10 µm.

Mitochondrial heteroplasmic cells can be obtained through both co‐culture and cell fusion methods, but cell fusion allows for a more uniform distribution of exogenous mitochondria within the cell (Figure 8A). Although the efficiency of xeno‐MT varies across different methods (Figure 8B), the impact on energy production and ROS elimination in TM4 cells is similar (Figure 8C,D). Furthermore, transplanting mitochondria from different types of donor cells produced similar effects on TM4 cells (Figure 8C,D). Comparative analysis showed that, across the endpoints tested here, allogeneic mitochondrial transplantation (Allo‐MT) and xenogeneic MT (Xeno‐MT) showed broadly similar directional effects in aged mice, including rotarod performance, ATP content, ROS levels, and mtDNA copy number in brain and muscle (Figure 8E–H). However, the present study was not designed or powered to establish donor equivalence across broader functional, molecular, or long‐term outcomes. Given the therapeutic insights from xeno‐MT‐induced mitophagy, we next asked whether progressively increasing room‐temperature exposure would differentially affect donor mitochondrial respiratory competence, membrane integrity, and biological efficacy. To address this, we generated Fresh‐mito, RT‐2h‐mito, RT‐6h‐mito, RT‐12h‐mito, and a fully disrupted mitochondrial fraction (Disrupted‐mito) for comparative characterization.

### 2.9 Progressive Loss of Respiratory Competence, Cytochrome c Responsiveness, and In Vivo Efficacy in Room‐Temperature‐Compromised Mitochondria

**FIGURE 9:**
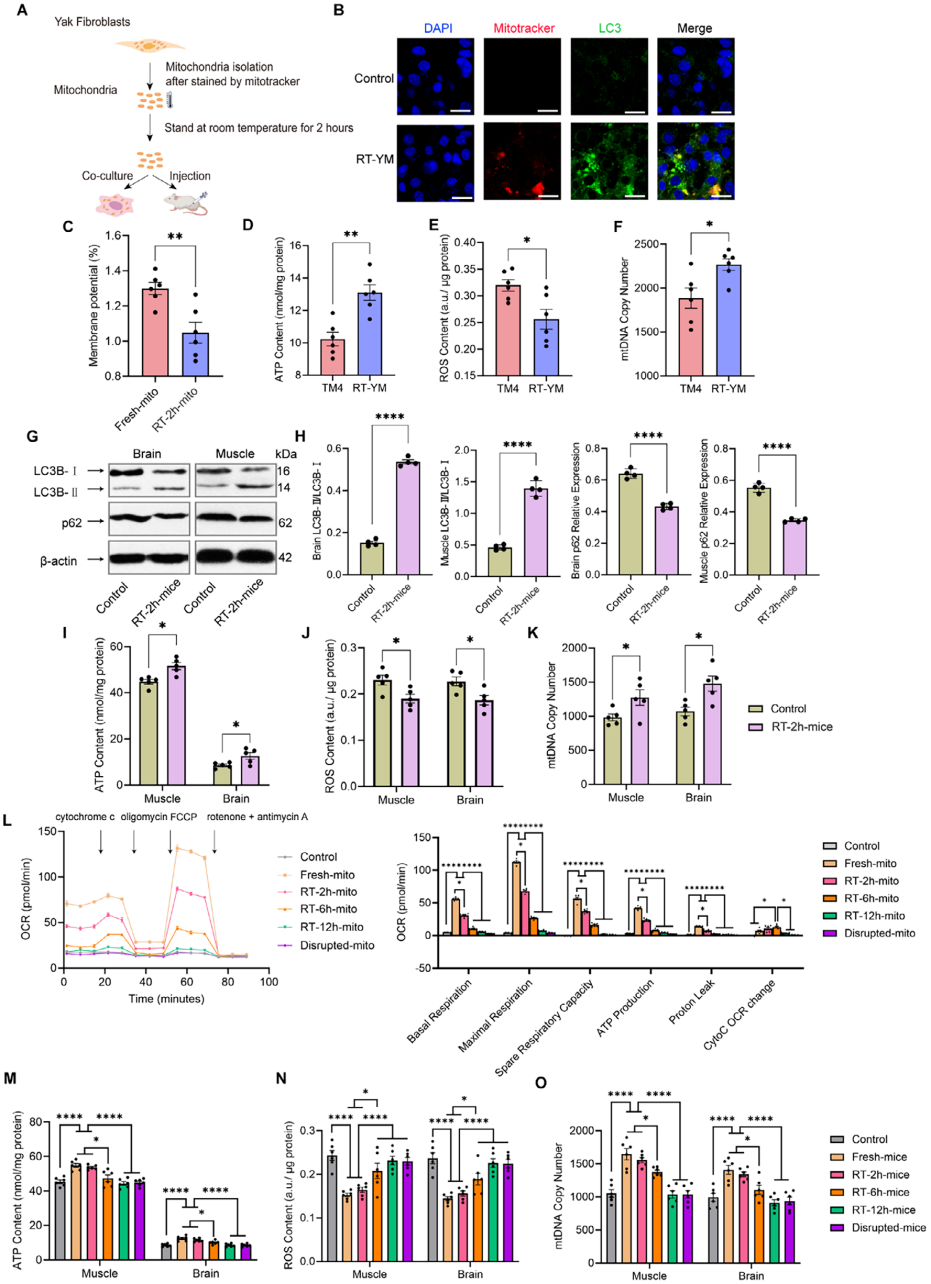
Progressive Room‐temperature Damage Reduces Donor Mitochondrial Function and in vivo Efficacy. (A) Schematic diagram of room‐temperature treatment of donor mitochondria and preparation of Fresh‐mito, RT‐2h‐mito, RT‐6h‐mito, RT‐12h‐mito, and Disrupted‐mito. (B) Immunofluorescence of LC3B+ autophagosomes in RT‐YM. (C) Mitochondrial membrane potential detection after storage at room temperature for 2 h. n = 6 mice per group. (D) ATP content measurement following the transplantation of RT‐2h‐mito in vitro. n = 6 mice per group. (E) ROS content measurement following the transplantation of RT‐2h‐mito in vitro. n = 6 mice per group. (F) mtDNA copy number detection after transplantation of RT‐2h‐mito in vitro. n = 6 mice per group. (G, H) Western blot detection of autophagic flux marker LC3B and autophagic substrate p62 in mouse brain and muscle following transplantation of RT‐2h‐mito. n = 4 mice per group. (I) ATP content in mouse brain and muscle following transplantation of RT‐2h‐mito. n = 5 mice per group. (J) ROS content in mouse brain and muscle following transplantation of RT‐2h‐mito. n = 5 mice per group. (K) mtDNA copy number in mouse brain and muscle after transplantation of RT‐2h‐mito. n = 5 mice per group. (L) Representative OCR traces and quantification of basal respiration, maximal respiration, spare respiratory capacity, ATP production, proton leak, and absolute OCR increase after cytochrome c addition. N = 6 independent experiments. (M–O) ATP content, ROS levels, and mtDNA copy number in brain and muscle from Control, Fresh‐mice, RT‐2h‐mice, RT‐6h‐mice, RT‐12h‐mice, and Disrupted‐mice. n = 5 mice per group. Fresh‐mito, freshly isolated donor mitochondria; RT‐Xh‐mito, donor mitochondria incubated at room temperature for X hours before transplantation. RT‐Xh‐mice, mouse received RT‐Xh‐mito. Disrupted‐mito denotes a fully disrupted mitochondrial‐enriched preparation generated by repeated freeze–thaw cycles and sonication, and was included to assess whether nonspecific debris‐like material could reproduce the effects observed with Fresh‐mito or partially compromised preparations. Disrupted‐mice denotes mouse received Disrupted‐mito. For RT‐YM cells, RT‐2h‐mito were incubated with TM4 cells for 12 h and applied at 100 µg/mL. Data are presented as mean ± SEM. For panels C–F, H–K, statistical significance was calculated via two‐tailed unpaired Student's *t*‐test. For panels L–O, Statistical significance was assessed by one‐way ANOVA followed by Tukey's multiple comparisons test.

RT‐2h‐mito were then transplanted into TM4 cells (RT‐YM), while Fresh‐mito, RT‐2h‐mito, RT‐6h‐mito, RT‐12h‐mito, Disrupted‐mito were transplanted into aged mice (Fresh‐mice, RT‐2h‐mice, RT‐6h‐mice, RT‐12h‐mice, Disrupted‐mice, respectively) (Figure 9A). As expected, RT‐YM obtained through co‐culture successfully activated autophagy (Figure 9B). Despite their damaged membrane potential (Figure 9C), the exogenous mitochondria were still able to improve energy production, ROS clearance, and mtDNA copy number in TM4 cells (Figure 9D–F). Similarly, Western blot analysis of autophagic markers LC3B and autophagy substrate p62 confirmed that RT‐2h‐mito activated autophagy in the brains and skeletal muscles of aged mice (Figure 9G,H), enhancing energy production, ROS clearance, and mtDNA copy number (Figure 9I–K).

OCR analysis showed that mitochondrial respiratory competence declined progressively with increasing room‐temperature exposure. Basal respiration, maximal respiration, spare respiratory capacity, ATP production, and proton leak were all highest in Fresh‐mito and decreased with prolonged room‐temperature incubation, with RT‐12h‐mito approaching a low functional plateau and Disrupted‐mito remaining near background levels (Figure 9L).

To further assess outer membrane integrity, we quantified the absolute OCR increase after cytochrome c addition. Fresh‐mito showed only a modest cytochrome c response, consistent with relatively preserved membrane integrity. RT‐2h‐mito exhibited a moderate recovery, and RT‐6h‐mito showed a stronger increase that was significantly higher than Control, Fresh‐mito, RT‐12h‐mito, and Disrupted‐mito, while remaining higher than RT‐2h‐mito without reaching statistical significance. RT‐12h‐mito still displayed a detectable but limited response while remaining close to a low respiratory floor, whereas Disrupted‐mito showed little meaningful recovery and the Control group remained largely unchanged (Figure 9L).

We then examined whether progressively damaged mitochondrial preparations retained in vivo efficacy after 5 times treatments. Aged mice receiving Fresh‐mito or RT‐2h‐mito showed significantly higher ATP production, lower ROS levels, and increased mtDNA copy number than RT‐6h‐mice, and both groups were markedly superior to RT‐12h‐mice, Disrupted‐mice, and Control mice. RT‐6h‐mice retained partial activity but consistently performed worse than RT‐2h‐mice. In contrast, RT‐12h‐mice and Disrupted‐mice were comparable to the Control group across these readouts (Figure 9M–O). Together, these results indicate that partial donor mitochondrial damage is compatible with retained activity, whereas more extensive respiratory and structural compromise markedly attenuates biological efficacy.

Although the present study did not include marker‐based organelle purity profiling of the commercial mitochondrial preparation, the failure of the fully disrupted fraction to reproduce the effects of Fresh‐mito or partially compromised preparations argues against a purely nonspecific debris‐driven explanation for the observed responses.

### 2.10 Systemic Improvements in Mitochondrial Function and Aging Markers in RT‐2h‐Mice

**FIGURE 10:**
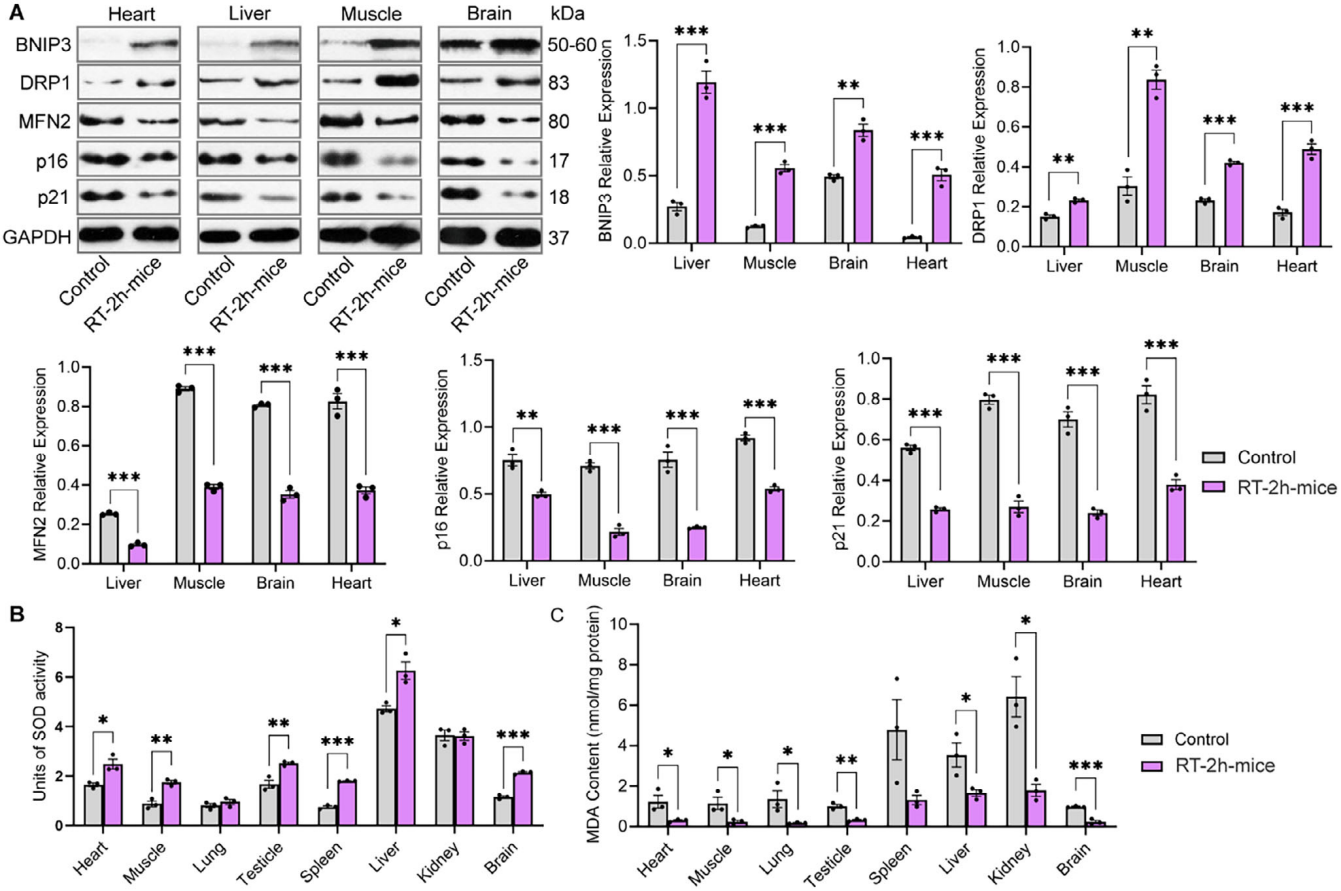
Changes in Mitochondrial Function and Aging‐associated Markers in RT‐2h‐mice. (A) The expression levels of mitophagy‐related proteins BNIP3 and mitochondrial dynamics proteins DRP1, MFN2 along with aging‐associated markers p16 and p21. n = 3 mice per group. (B) SOD activity of various organs in RT‐2h‐mice. n = 3 mice per group. (C) MDA level of various organs in RT‐2h‐mice. n = 3 mice per group. Statistical significance for panel A was assessed within each tissue and protein using the Mann–Whitney *U* test. For panels B and C, statistical significance for each tissue was assessed using the Mann–Whitney *U* test. For tissue‐wise comparisons within each endpoint family, p values were additionally adjusted using the Benjamini–Hochberg procedure. Data are expressed as mean ± SEM. ^*^ *p* < 0.05, ^**^ *p* < 0.01, ^***^ *p* < 0.001.

After confirming that exogenous mitochondria with disrupted membrane potential can still activate mitophagy‐related responses, enhance energy production, and reduce ROS levels, we next examined the impact of RT‐2h‐mito transplantation on the organs most vulnerable to aging, including the heart, liver, skeletal muscle, and brain. In RT‐2h‐mice, transplantation of RT‐2h‐mito significantly increased protein levels of BNIP3, a key mitophagy‐related regulator, along with upregulation of the mitochondrial fission protein DRP1 and downregulation of the fusion protein MFN2. Expression of aging‐associated markers p16 and p21 was markedly reduced across these tissues (Figure 10A). Furthermore, RT‐2h‐mice exhibited enhanced antioxidant capacity, as indicated by increased superoxide dismutase (SOD) activity (Figure 10B) and markedly reduced malondialdehyde (MDA) levels (Figure 10C) in multiple organs.

## 3 Discussion

Our findings indicate that xeno‐MT is associated with improvement in selected age‐associated phenotypes and mitochondrial functional readouts, together with changes in host mitochondrial quality‐control‐related pathways. The present data support functional involvement of lysosome‐dependent autophagic flux, but do not establish pathway‐specific dependence on PINK1‐Parkin or any single mitochondrial dynamics regulator. The phenomenon of mitochondrial transfer into cells was first discovered in 1982 [10], and it took decades for researchers to realize its universality after considerable controversy. Cell‐free mitochondria are a rich and normal component of blood, indicating that human or animal physiology permits the existence of cell‐free mitochondria [16]. The use of autologous or heterologous cell‐free mitochondria may be well tolerated [7]. Although reports on using MT to combat aging or improve mitochondrial dysfunction diseases are increasing [17], the underlying mechanisms remain incompletely understood, and the therapeutic effects are still debated [18]. While there has been some progress in the research on xeno‐MT [19, 20, 21], most studies have focused on the transplantation of human mitochondria in animal models, such as mice. However, a widely applicable xenogeneic mitochondrial source has not yet been identified for clinical practice. Therefore, our study selected yak mitochondria as the donor source, aiming to fill this gap and provide additional proof‐of‐concept evidence supporting the biological activity of yak‐derived xeno‐MT in the present experimental setting. In the limited comparative setting examined here, xeno‐MT showed broadly similar directional effects to Allo‐MT across the endpoints assessed. However, the present study was not designed or powered to establish donor equivalence across broader functional, molecular, or long‐term outcomes.

An important caveat is that the in vivo intravenous transplantation model and the in vitro fusion‐based heteroplasmic model involve distinct routes of mitochondrial entry. Free mitochondria delivered extracellularly must be taken up by recipient cells, whereas the fusion model places donor mitochondria directly into the intracellular environment [22, 23] These differences may influence the timing, trafficking, and quality‐control pathways engaged by exogenous mitochondria. Accordingly, the in vitro findings should be interpreted as supportive of intracellular mitochondrial quality‐control responses rather than as a direct mechanistic surrogate for the in vivo uptake process. Exogenous mitochondria enter host cells, where they act as xenogeneic stimuli that trigger a controlled host response without inducing harmful systemic inflammation, as evidenced by the absence of significant changes in white blood cell counts, neutrophil percentages, lymphocyte percentages, IL‐6 and TNF‐α levels, alongside a modest, non‐significant elevation of the immunoregulatory cytokine IL‐10. In the present study, safety evaluation was limited to short‐term hematologic indices and a small panel of serum cytokines, and no overt abnormalities were observed under these conditions. However, these data do not address chronic immune activation, autoimmunity, tumorigenesis, or long‐term reproductive and germline consequences. In middle‐aged females treated during pregnancy, offspring from the xeno‐MT group showed greater postnatal weight gain, whereas the number of live‐born pups per litter was not significantly different between groups. Given the detection of exogenous mtDNA in offspring, these findings should be interpreted cautiously and should not be taken as evidence of reproductive safety or benefit. Dedicated studies will be required to clarify maternal‐fetal transfer, germline relevance, and the biological basis of this observation. Accordingly, the present findings should be viewed as proof‐of‐concept evidence of biological activity and short‐term tolerability, rather than as establishing translational readiness.

Exogenous mitochondria likely act as a stimulus that engages host mitochondrial quality‐control responses without overt systemic inflammation. In aged tissues, xeno‐MT increased LC3B‐II/LC3B‐I, altered p62, and was accompanied by changes in BNIP3 and mitochondrial dynamics‐related proteins. Importantly, the CQ intervention data show that blocking lysosome‐dependent autophagic flux attenuates the beneficial effects of xeno‐MT on ATP production, ROS clearance, and mtDNA copy number. These findings support a functional contribution of autophagic flux to the mitochondrial benefits of xeno‐MT. At the same time, our current data do not establish pathway‐specific dependency on PINK1‐Parkin in vivo. Therefore, the involvement of PINK1‐Parkin‐related mitophagy should be interpreted as a mechanistically supported hypothesis. Similarly, the observed changes in BNIP3, BCL2, and mitochondrial fission/fusion proteins are most appropriately viewed as evidence consistent with mitochondrial quality‐control remodeling.

ddPCR offers unparalleled sensitivity, absolute quantification, and high tolerance to inhibitors, making it an ideal tool for precise and high‐throughput detection in genomic and molecular research [24]. Using ddPCR, we detected exogenous mtDNA across multiple tissues, including brain and skeletal muscle, indicating broad biodistribution and short‐term persistence after transplantation. Aged mice retained lower levels of exogenous mtDNA in several tissues than young or middle‐aged mice. However, the present study cannot distinguish whether these age‐dependent differences reflect reduced uptake, accelerated clearance, enhanced degradation, or a combination of these processes. These distribution data should therefore be interpreted descriptively and not as direct evidence of tissue‐specific mitophagy intensity or therapeutic relevance to specific disease contexts.

Mitochondria act a crucial role in providing the essential energy for sperm movement and the production of steroid hormones by getting in the act of mitochondrial ATP synthesis [25, 26, 27]. Thus, it is not surprising that xeno‐MT increased the total amount of motile sperm and beat frequency in middle‐aged male mice in the present study. These findings suggest potential relevance to sperm motility in the present experimental setting. Moreover, mitochondrial dysfunction is one of the significant cytoplasmic changes in elderly oocytes, leading to a decrease in cytoplasmic capacity and oocyte maturation, providing energy for the formation of spindles and the separation of chromosomes [28, 29] In this study, we injected exogenous mitochondria to 12‐month‐aged female mice (actually old enough for reproduction) during pregnancy. In middle‐aged females treated during pregnancy, offspring from the xeno‐MT group showed greater postnatal weight gain, whereas the number of live‐born pups per litter did not differ significantly between groups. Because exogenous mtDNA was also detected in offspring, these findings require particularly cautious interpretation. The present data do not establish benefit, safety, maternal‐fetal transfer mechanism, or germline relevance, and dedicated follow‐up studies will be required to clarify the biological basis of this observation.

The age‐dependent variation in exogenous mtDNA retention likely reflects the natural decline in mitochondrial turnover and quality control efficiency that accompanies aging. Tissues from younger individuals, characterized by more active mitochondrial dynamics and efficient mitophagy, may process and clear exogenous mitochondria at a different rate or through distinct pathways compared to aged tissues, where these homeostatic mechanisms are attenuated. This pattern underscores a fundamental principle: the host's intrinsic mitochondrial fitness critically shapes the fate and impact of transplanted organelles. The corresponding organ‐ and age‐specific differences in bioenergetic outcomes—such as ATP levels, ROS accumulation, and mtDNA copy number—further demonstrate the context sensitive nature of xeno‐MT. Notably, the most pronounced functional improvements occurred in aged organs exhibiting the most severe baseline mitochondrial dysfunction. Rather than representing inconsistency, this variability highlights a therapeutic strength: xeno MT appears to preferentially restore function in tissues with the greatest need, consistent with its host‐directed mechanism of action. The coordinated increase in ATP and mtDNA copy number, alongside the reduction in ROS—particularly evident in aged animals—will be discussed as collective evidence that the treatment successfully triggers a program of enhanced mitochondrial biogenesis and renewal within the host.

In the realm of mtDNA replication, the LSP and O_H_ on the light strand are critical for complete mtDNA replication [30]. Consequently, we also assessed CHG and CHH methylation levels. The observed lower methylation levels in CpG, CHG, and CHH in the muscle tissue of xeno‐MT‐treated aged (A) mice may explain their higher mtDNA copy number compared to aged control (AC) mice. However, the absence of significant differences in methylation levels in the brain between A and AC mice indicates a distinct mechanism driving the increased mtDNA copy number in the brain of A mice. Additionally, the brain and muscle tissues of A mice consistently exhibited the lowest methylation levels across all groups (A, AC, young (Y), and young control (YC)). Previous studies have also noted a decline in D‐loop methylation levels with age [31], suggesting that reduced methylation may compensate for heightened mtDNA dysfunction in older mice. Furthermore, a significant inverse correlation between mtDNA copy number and methylation levels substantiates the regulatory impact of LSP and O_H_ methylation on mtDNA copy number in aged mice. While the inverse relationship between D‐loop methylation and mtDNA copy number is well‐documented [32], our findings reveal that, beyond the conventional CpG pattern, CHG and CHH methylation are also pivotal to mtDNA replication.

Genome‐wide, SNPs and InDels accumulate with age, leading to various diseases [33]. This was validated in our study with aged and young mice. However, after xeno‐MT treatment, the significant SNPs and InDels between A and AC groups in genes clustered to cognition, movement, and other functions. This may be because xeno‐MT changes the methylation of genes in specific signaling pathways (mTOR and AMPK), these pathways are closely linked to DNA repair mechanisms and maintain cellular health [34, 35]. Additionally, in aged mice, common mtDNA deletions occur within the 4236 bp region between positions 8884 and 13 120. These deletions, which accumulate with age, ultimately lead to mitochondrial dysfunction diseases and show a significant correlation with aging [36]. Besides, we found that the intergroup differences in the 9500–10 500 region were the greatest, regardless of whether it was the number of SNPs or indels. The colocalization analysis of exogenous mitochondria with PRKN in heteroplasmic cells, together with the progressive decrease in exogenous mitochondrial DNA copy number across generations, is consistent with mitophagy‐associated clearance of exogenous mitochondria. However, because it is difficult to distinguish between exogenous and endogenous mitochondria, the latter cannot be stained for labeling and colocalizing with mitophagy‐related markers. Therefore, we selected mitochondrial genomes from the whole‐genome sequencing data and performed sliding window analysis. This revealed a reduction in defective mitochondria (mitochondrial DNA mutations/fragments‐deleted mitochondria) in the treatment group of mice, which indicates that endogenous mitochondria are cleared. The mitochondrial DNA fragments (9500–10 500) identified are common deletion/mutation regions in aged mice, encompassing the *ND3*, *ND4*, and *ND4L* genes. These genes encode subunits of complex I, and their deletion or mutation may impair complex I assembly, disrupt proton pumping, and reduce the capacity to maintain an effective transmembrane potential gradient. Such membrane‐potential disturbance is a known upstream signal for PINK1‐Parkin‐related mitophagy, but was not directly demonstrated in vivo here. Thus, the reduction in mtDNA variants after xeno‐MT is consistent with enhanced mitochondrial quality control affecting host's defective mitochondrial populations. The results of demethylation of LSP and O_H_ as well as decrease of mitochondria with mtDNA deletions suggest that xeno‐MT treatment induces the promote of mitochondria replication with intact mtDNA, resulting in enhanced respiratory function.

Combined transcriptome and whole‐genome methylation analysis, except for the phagosome‐related genes *Mrc2* and *Itgb2* in the muscle, the hypomethylated and upregulated gene *Lrp1* acts as a positive mediator in the activation of autophagy [37], while *Capn6* reduces autophagy by maintaining mTOR signal transduction [38]. In the brain, the hypermethylated and downregulated gene *Prkcd* was reported to suppress autophagy by involving in mTOR pathway [39]. This evidence suggested that the exogenous mitochondria are initially recognized as foreign substances after xeno‐MT, leading to significant changes in the expression and methylation of phagosome‐ and autophagy‐related genes. Because young‐control baselines were not included in the RNA‐seq and WGBS experiments, these datasets cannot distinguish rejuvenation‐like normalization from other treatment‐associated responses, including stress‐related remodeling. *Prkn* has been extensively studied as a component of PINK1‐Parkin‐mediated mitophagy. In this pathway, PINK1‐dependent phosphorylation events can activate the cytosolic E3 ubiquitin ligase PRKN and promote ubiquitination of outer mitochondrial membrane proteins, thereby facilitating the recruitment of autophagy receptors such as SQSTM1/p62, OPTN, and MAP1LC3/LC3 to damaged mitochondria [40, 41, 42]. In the present study, *Prkn* was identified in the WGS/WGBS analyses, with altered intronic methylation and accompanying SNP/InDel signals after xeno‐MT treatment. However, *Prkn* transcript levels were not increased, and we did not directly measure PINK1 stabilization, PRKN phosphorylation, mitochondrial PRKN recruitment, ubiquitination of mitochondrial outer‐membrane substrates, or pathway dependency using genetic or targeted pharmacological perturbation. Therefore, these *Prkn*‐associated genomic and epigenetic changes should be interpreted as candidate signals potentially related to mitochondrial quality‐control remodeling, rather than as direct evidence of PINK1‐Parkin pathway activation in vivo. In the fusion‐based heteroplasmic cell model, immunofluorescence findings were consistent with PINK1‐Parkin‐related mitochondrial quality‐control responses after intracellular xenogeneic mitochondrial entry, but this model is mechanistically distinct from in vivo free‐mitochondria transplantation. Similarly, previous studies have linked BNIP3 and BCL2 to autophagy regulation and reported interactions between BNIP3 and Parkin/PINK1‐related mitophagy in specific cellular contexts [43, 44, 45]. In our study, the observed changes in BNIP3, BCL2, and mitochondrial dynamics‐related proteins are therefore best viewed as supportive evidence of broad mitochondrial quality‐control remodeling after xeno‐MT, but they do not establish pathway‐specific dependence on PINK1‐Parkin.

To extend our observations beyond the primary yak‐to‐mouse setting, we conducted complementary experiments using additional donor contexts. These data suggest that beneficial effects are not restricted to a single donor source. However, the current study provides the most detailed in vivo evidence for yak‐derived xeno‐MT, and the cross‐source comparisons remain limited in scope and endpoint coverage. Therefore, our results should be interpreted as supporting the feasibility of multiple donor contexts rather than establishing broad donor equivalence or a fully donor‐agnostic therapeutic framework.

We further characterized donor mitochondria subjected to progressive room‐temperature damage and found that donor mitochondrial integrity is not an all‐or‐none determinant of activity. Fresh‐mito showed the strongest respiratory competence and the most robust in vivo efficacy. RT‐2h‐mito and RT‐6h‐mito retained partial OCR and partial biological activity, whereas RT‐12h‐mito approached a low functional plateau and the fully disrupted fraction remained near background levels. Importantly, the fully disrupted mitochondrial fraction failed to reproduce the beneficial effects observed with Fresh‐mito or partially compromised preparations, arguing against a purely debris‐ or DAMP‐driven explanation for the observed responses. The cytochrome c response was most evident in moderately damaged mitochondrial preparations, particularly RT‐6h‐mito, whereas severely damaged samples showed only limited recovery, consistent with collapse of broader respiratory competence beyond outer membrane injury alone. In vivo, Fresh‐mice and RT‐2h‐mice showed the strongest improvement in ATP production, ROS clearance, and mtDNA copy number, whereas RT‐6h‐mice retained only partial efficacy, and RT‐12h‐mice and Disrupted‐mice were comparable to Control mice. Together, these findings indicate that partial donor mitochondrial damage is compatible with retained activity, but both respiratory competence and in vivo efficacy decline progressively with more extensive structural and bioenergetic impairment. Our study shows that xeno‐MT improves selected age‐associated phenotypes and is accompanied by broad remodeling of host mitochondrial quality‐control‐related pathways, rather than being explained solely by direct donor bioenergetic supplementation.

## 4 Limitations

Several limitations of the present study should be acknowledged. First, although CQ‐based pharmacological interference supports a functional contribution of lysosome‐dependent autophagic flux, the present data do not establish pathway‐specific dependence on PINK1‐Parkin or any individual mitochondrial quality‐control regulator. Second, the fusion‐based heteroplasmic cell system and the in vivo intravenous transplantation model involve distinct routes of mitochondrial entry and therefore should not be interpreted as mechanistically identical. Third, because young‐control baselines were not included in the RNA‐seq and WGBS datasets, these analyses cannot distinguish rejuvenation‐like normalization toward a youthful molecular state from other treatment‐associated responses and should therefore be interpreted primarily as candidate‐discovery datasets within the aged background. Fourth, safety evaluation in the present study was limited to short‐term hematologic indices and a restricted serum cytokine panel, and therefore does not address chronic immune activation, autoimmunity, tumorigenesis, or long‐term reproductive and germline consequences. The detection of exogenous mtDNA in offspring should likewise be interpreted cautiously as an observation requiring dedicated follow‐up rather than as evidence of safety or benefit.

## 5 Conclusion

In summary, our study shows that xeno‐MT improves several selected age‐associated phenotypes and mitochondrial functional readouts in mice. These findings support the relevance of xeno‐MT to age‐associated mitochondrial dysfunction and related phenotypes under the experimental conditions tested. However, broader mechanistic and translational conclusions remain premature, and long‐term safety, donor‐source generalizability, and pathway‐specific dependence will require dedicated future investigation.

## 6 Experimental Section/Methods

### 6.1 Animals and Experimental Groups

Different ages of C57BL/6J mice were recruited in this study including aged group (18 month‐old, female), middle‐aged group (12‐month‐old, male and female) and young group (3 month‐old, female). Animals were accommodated under identical conditions, enjoying unrestrained access to standard laboratory mouse nourishment and water, while situated in an environment maintained at 25°C and a 12 h dark/light cycle. The experimental and animal care protocols were approved by the China Agricultural University Laboratory Animal Welfare and Animal Experimental Ethical Inspection.

Different ages of female mice were randomly and averagely divided into two subgroups, which one subgroup named experimental group, including young group (Y), middle‐aged group (M) and aged group (A), and another subgroup named control group (YC, MC, AC, respectively), each group contained 10 animals. The mice in each experimental group (Y, M, A) received an intravenous (i.v.) injection of the commercially isolated mitochondria‐enriched preparation derived from yak cells (10 mg/kg body weight) suspended in saline solution, administered once every two days for a total of ten times, and control groups (YC, MC, AC) mice were administered an equivalent volume of saline. Mice in A, AC, M, MC, Y and YC groups were euthanized with 10% pentobarbital sodium after 7 days’ injection. Afterward, the mice were transcardially perfused with ice‐cold PBS to move out blood, and then mice heart, liver, spleen, lung, kidney, ovary, stomach, brain, hypothalamus and skeletal muscle were dissected out and stored at −20°C for further investigation. Besides, male and female mice (12 months old) were prepared to evaluate the reproductive capacities. Shortly, 10 males (S) and 10 females (F) were chosen at random, and the MT protocol was executed by intravenous injection. For males, sperm motility was evaluated at 7 days after injection. The testicles were also collected for evaluation of mitochondrial DNA (mtDNA) copy number. For females, injections were initiated on the second day after cohabitation of the female and male mice, and the appearance of a vaginal plug in the female, continuing until the progeny birth. After birth, the number and growth curve were recorded. Same number of male and pregnant female mice served as controls (SC and FC).

The every‐2‐day, 10‐dose regimen was selected to provide repeated systemic exposure over a sustained treatment window while avoiding overly dense handling/injection intervals.

### 6.2 Cell Line and Isolation of Mitochondria

Exogenous mitochondria designated for injections were procured from yak ear fibroblasts cultured in our laboratory. The cells were digested with 0.25% trypsin/EDTA at 37°C for 2 min, then washed twice with PBS. Mitochondria isolation was performed according to the manufacturer's protocol of mitochondrial isolation kit (C3601, Beyotime, China). Briefly, the cells were homogenized in cold isolation solution, and then centrifuged at 600 g for 10 min at 4°C to eliminate contaminants and sizable organelles. The supernatant was collected, resuspended in the isolation solution, and centrifuged again at 11 000 g for 10 min at 4°C. The mitochondrial precipitate was washed 2 times with isolation solution and stored at 4°C for subsequent utilization. Mitochondrial concentration determination employed the BCA method.

Mitochondria were isolated using a commercial kit and are therefore referred to throughout the manuscript as a mitochondria‐enriched preparation rather than a fully purified organelle fraction.

### 6.3 Phenotype Evaluation of Mice

In this study, the rotarod test was designed as a behavioral indicator to evaluate mouse fatigue tolerance. Throughout the experiment, the speed was maintained at 40 rpm, and the duration each animal remained on the rod was documented as the latent period. Each mouse underwent four consecutive trials with 10 min rest intervals to preclude stress and fatigue, and the maximal time on the rod was selected for analysis. Additionally, aging parameters were scored to assess signs of aging in elderly mice, employing a scale ranging from 0 (none) to 3 (high) [46]. Whole blood was collected via retro‐orbital bleeding for complete blood count analysis.

Caudal epididymides of S and SC mice were collected from both sides and placed into two EP tubes containing 200 µL of preheated (37°C) sperm capacitating fluid. The cauda epididymis was punctured with a syringe needle, incubated in a 37°C‐water bath for 15 min to fully release sperm. Sperm suspension of 10 µL was dripped onto a special slide on the microscope platform at 37°C, followed by covering with a cover slide. Sperm movement was recorded by CASA analysis system, and various indexes were analyzed. CASA analysis system (Version 1 ceros, Hamilton Thorne Research) parameter Settings are as follows: minimum contrast 50, the minimum cell size 4 pixels, sampling frequency is 60 Hz. At least 500 sperm were counted in each sample.

When xeno‐MT and control mice give births, the live birth rate (the number of live‐born pups per litter) was recorded. To construct growth curves, newborn mice were weighed and subsequent weekly measurements until they reached 28 days. Three separate cohorts of animals were utilized. In addition, we employed nest‐PCR to quantify exogenous mtDNA in the muscle tissue of pups. Two primer pairs (nPCR‐1 and nPCR‐2) were designed to detect the exogenous mtDNA in muscle of the progeny based on the yak mtDNA sequence (GenBank: KU891851), the sequences of primers were listed in Table S2 The reaction mixture consisted of 12.5 µL PCR Supermix, 1 µL of F1 and R1 primers (100 µm each), 2 µL of the template DNA, and ddH_2_O to bring the total volume to 25 µL. For the secondary PCR reaction, 5 µL of the primary PCR product was used, and the remaining components were identical. Both primary and secondary amplification protocols followed these steps: initial denaturation at 95°C for 3 min, followed by 35 cycles of denaturation at 95°C for 10 s, annealing at 53°C for 15 s, and extension at 72°C for 30 s, concluding with a final extension at 72°C for 5 min. Droplet digital PCR was employed to detect the exogenous mtDNA, the procedure was identical to 5.6.

### 6.4 Biochemical Assays on Mouse Tissues

Levels of ATP and ROS in various mouse cells and tissues (heart, liver, spleen, lung, kidney, ovary, stomach, brain, hypothalamus, and skeletal muscle) were measured using commercial kits (Beyotime; ATP Assay Kit, S0026; ROS Assay Kit, S0033S). ATP content was determined via a spectrophotometric method, while ROS levels were assessed using DCFH DA fluorescence. In addition, total SOD activity and MDA content were evaluated using the Total SOD Activity (NBT Method) Assay Kit (S0101, Beyotime, China) and the Lipid Peroxidation (MDA) Assay Kit (S0131, Beyotime, China), respectively, following the manufacturer's instructions.

### 6.5 ElisaElisa

An enzyme‐linked immunosorbent assay (ELISA) was performed using mouse serum. Mouse IL‐ 6, IL‐10, and TNF‐α levels were measured in the mouse serum samples using commercially available ELISA kits: mouse IL‐6 ELISA kit (ab222503, Abcam), mouse IL 10 ELISA kit (ab255729, Abcam, USA), and mouse TNF‐α ELISA kit (ab208348, Abcam, USA).

### 6.6 Distribution of Exogenous Mitochondria

CellLight mitochondria‐GFP (C10600, ThermoFisher, USA) were used to label mitochondria. Saline solution containing fluorescently labeled mitochondria was administered to two mice (one male and one female) via i.v. tail vein injection at a dosage of 10 mg/kg body weight. Following a 2 h post‐injection period, the mice were humanely euthanized using 10% pentobarbital sodium. Subsequent to euthanasia, transcardial perfusion with PBS (0.01 m, pH 7.4) was performed to remove blood. Organs including heart, liver, spleen, lung, kidney, ovary, testis, stomach, brain, hypothalamus and skeletal muscle were meticulously excised and immediately fixed in 4% paraformaldehyde. Thereafter, frozen sections (30 µm) of the tissues were prepared using a cryomicrotome (Leica, Germany). Fluorescent assay was performed on tissue sections to confirm the uptake of exogenous mitochondria into the cells.

### 6.7 Quantification of Exogenous mtDNA by Droplet Digital PCR (ddPCR)

Tissue DNAs were extracted according to the manufacturer's instruction of universal genomic DNA Kit (Aidlab, Beijing, China). Before ddPCR was carried out, the genome DNA of each sample was diluted to the concentration of 100 ng/µL. Based on the mitochondrial genome sequence of Bos grunniens listed in GenBank (KU891851), the sequence of NADH dehydrogenase subunit 1 (ND1) gene was analyzed using Primer Premier 5.0 software. One probe (ND1‐probe) and a pair of specific primers (ddPCR‐ND1) were prepared subsequently. The primer and probe sequences were listed in Table S1. After the reaction system and the annealing temperature optimization, the reaction system was as follows: ddPCR Supermix for probe (no dUTP) 10 µL, F and R primer (10 µm) 1.8 µL respectively, probe (10 µm) 0.5 µL, 2 µL of the template, the system was added up to 20 µL using ddH2O. The nucleic acid content of each sample added in the system should not exceed the specified detection range (1 to 20 000 copies of complete genomic DNA). Then, 20 µL of the sample reaction system was placed in 8 wells in the middle row of the DG8 cartridge, and 70 µL of oil was added to the DG8 cartridge oil wells to form an oil barrier. Subsequently, the pad was covered and then placed in the droplet generator to generate droplets. We placed the resulting droplets in a 96‐well plate and sealed them in a PX1 heat sealer. And then PCR amplification was performed. The amplification procedure was as follows: 95°C for 10 min, followed by 40 cycles of 94°C for 30 s, 60°C for 60 s and 98°C for 10 min. After amplification, the 96 well plate was placed in a micrometer reader (QX200) for data reading. To absolutely quantify target DNA, data were analyzed using the QXManager 1.2 Standard Edition Software (Bio‐Rad) and expressed as copies/µL in ddPCR reactions.

### 6.8 Absolute Quantification of mtDNA Copy Number and Relative Telomere Length on Mouse Tissues

By referring to the copy number of the diploid nuclear genome, the mitochondrial genome copy number was determined by quantifying a fragment of *ND1* by qPCR method (LightCycler480 II, Roche, Rotkreuz, Switzerland). Nuclear DNA content was determined by amplification of a segment of a single copy gene, *GAPDH*. In detail, primers for 126‐bp *ND1* (qPCR‐ND1) and 85‐bp *GAPDH* (qPCR‐GAPDH) were designed based on the GenBank sequences DQ106412 and GU214026, respectively (Table S1). Amplicons were generated using routine PCR procedures. The PCR products were purified and cloned into the pMD 18‐T vector (Sangon Co., China). The standard samples were 10‐fold serially diluted from 107 to 102 copy numbers, which were used in absolute quantity for determining the mtDNA copy numbers per cell in each sample. The mtDNA copy number per cell in mice was computed by taking twice the ratio of the *ND1* and *GAPDH* values.

For aged mice, the relative telomere length of brain and muscle was evaluated using Relative Mouse Telomere Length Quantification qPCR Assay Kit (M8908, ScienceCell, USA).

### 6.9 Methylation Analysis of mtDNA LSP and O_H_ Region

In this study, we utilized the EZ‐96 DNA Methylation Gold Kit (Zymo Research, CA, USA) to perform bisulfite conversion on 20 µL of extracted genomic DNA, adhering to the manufacturer's specifications. The sequences of the target genes were obtained from GenBank (Accession No. M13875.1). Primers targeting these regions were crafted using Primer Premier 5.0, specific PCR procedures were shown in Table S3. The bisulfite‐treated DNA fragments were subsequently sequenced on the Illumina MiSeq platform (San Diego, CA, USA), employing high‐throughput, second‐generation sequencing technology. Post‐sequencing, raw data were subjected to adaptor and primer removal using Cutadapt 4.9, followed by merging at the R1 and R2 ends using PEAR 0.9.8. Quality assessment of the sequences was conducted using FastQC 0.11.9. Alignment of the trimmed and quality‐controlled sequences to the mouse mitochondrial genome (GenBank: DQ106412) was achieved using Bismark 0.22.3, utilizing a single‐end mapping approach. Methylation levels at CpG, CHG, and CHH (H = A, T, or C) sites were quantified by calculating the proportion of methylated cytosines relative to the total number of cytosines, both methylated and unmethylated. The Spearman method was employed to investigate the correlation between methylation levels at the LSP and OH regions (CpG, CHG, and CHH) and mtDNA copy number in the brains and muscles of aged (A and AC) and young (Y and YC) mice.

### 6.10 Genome Sequencing and Variant Calling

Genomic DNA was isolated from brain and muscle of A, AC and YC mice (N = 3) using the DNeasy Blood & Tissue Kits (Qiagen, Germany). Whole‐genome resequencing was performed on the DNBSEQ platform using 200–300 bp DNA libraries. The library construction involved the following steps: the qualified genomic DNA samples were randomly sheared into specific‐sized fragments using a Covaris instrument; target fragments were selected using Agencourt AMPure XP‐Medium kit to concentrate bands around 200–300 bp, and purified DNA was quantified with Qubit dsDNA HS AssayKit; end repair, A‐tailing, and adapter ligation were performed with the reaction mixture under controlled conditions; the ligation products were amplified by PCR and purified using Agencourt AMPure XP‐Medium, and checked with Agilent 2100 Bioanalyzer; the amplified products were denatured to single‐stranded and circularized, and un‐circularized DNA was digested to obtain the final library; the library quality was assessed using Agilent 2100 Bioanalyzer. The sequencing involved linear amplification (LM‐PCR) of the library, followed by single‐strand separation and circularization, and rolling circle amplification (RCA) to generate DNA Nano Balls (DNBs), which were then sequenced, the sequencing depth was 30x. The DNBSEQ platform uses optimized combinatorial probe‐anchor synthesis (cPAS) and enhanced DNA Nano Ball (DNB) core sequencing technology to achieve high‐throughput sequencing, ensuring data quality for each sample. DNA molecules were anchored and polymerized on nanospheres, and the high‐resolution imaging system captured light signals which were digitized to obtain the sequencing reads. Linear amplification of DNBs improved signal strength and reduced single‐copy error rates, matching DNB size with the active site size on the chip to enhance sequencing accuracy and efficiency. The raw image data were converted to raw sequence data (paired‐end reads) in FASTQ format by the DNBSEQ base calling software.

Quality control and filtering were performed on the raw data to ensure data quality before analysis. Contaminated reads with adapters, excessive Ns, or low‐quality bases were removed, resulting in clean reads for downstream analysis. Quality control and filtering were conducted using BGI's SOAPnuke (Version: v2.2.6) [47] with parameters: SOAPnuke filter −n 0.001 −l 10 −q 0.5 −Q 2. Further quality control of the filtered data included assessing base composition and quality distribution. The clean data were aligned to the reference genome using BWA (Version: V0.7.17) [48] with the mem algorithm, generating SAM files, which were then processed into sorted BAM files using Samtools (Version: V1.9) [49] with sort, fixmate, and markdup tools. Quality metrics such as genome alignment rate and coverage depth were assessed with Qualimap2qualimap (Version: V2.2.1) [50]. SNP and Indel detection and annotation were conducted using GATK (Version: V4.2.6.1). The process involved: generating GVCF files for each sample using HaplotypeCaller; merging GVCF files with CombineGVCFs; extracting variants with GenotypeGVCFs; separating SNPs and Indels with SelectVariants; applying quality control filters with VariantFiltration; and annotating variants with snpeff (Version: V4.3.1) [51] based on their genomic positions.

### 6.11 RNA‐Sequencing Analysis

Total RNA was isolated from brain and muscle of A and AC mice (N = 3) using RNA Easy Fast Tissue/Cell Kit for RNA‐seq analysis. cDNA library construction and sequencing were performed by the Beijing Genomics Institute using BGISEQ‐500 platform. High‐quality reads were aligned to the mouse reference genome GRCm39 using Bowtie2. Expression levels for each of the genes were normalized to fragments per kilobase of exon model per million mapped reads (FPKM) using RNA‐seq by Expectation Maximization (RSEM). We identified DEGs (differential expressed genes) between samples and performed clustering analysis and functional annotation. Genes with ≥2‐fold change and false discovery rates (FDR) of ≤0.001 were considered to be statistically significant. Pathways overrepresented by DEGs were annotated in the KEGG (Kyoto Encyclopedia of Genes and Genomes) database.

### 6.12 Whole Genome Bisulfite Sequencing

Genomic DNA was isolated from brain and muscle of A and AC mice (N = 3) using the DNeasy Blood & Tissue Kits (Qiagen, Germany). Whole‐genome bisulfite sequencing (WGBS) was performed starting with genomic DNA sheared to 200–300 bp fragments. The DNA fragments underwent end repair, A‐tailing at the 3’ ends, and adapter ligation. The bisulfite treatment was then conducted using the ZYMO EZ DNA Methylation‐Gold kit (Zymo Research, USA). Following desalting, gel extraction, and size selection of library fragments, PCR amplification was performed, followed by a second round of size selection. The constructed libraries were quality‐checked and those passing quality control were sequenced. Initial quality control of DNA samples was conducted to ensure sample integrity before proceeding with bisulfite library construction. After library preparation, the quality of the libraries was assessed again, and only those meeting the quality standards were sequenced. Data filtering was performed using BGI's SOAPnuke software, which involved removing adapter‐contaminated reads, reads with more than 1% unknown bases (Ns), and low‐quality reads (reads where more than 40% of bases had a quality score below 10). The resulting clean reads were saved in FASTQ format. Sequence alignment was performed using Bismark, aligning the clean reads to the reference genome and calculating alignment rate and bisulfite conversion rate. Duplicate reads were removed using Bismark's deduplicate_bismark program with default parameters, and methylation information was extracted at single‐base resolution.

### 6.13 Autophagic Flux Analysis

To assess whether xeno‐MT induced active autophagic flux in aged tissues, four experimental groups were established: untreated control, xeno‐MT alone, xeno‐MT followed by chloroquine (CQ), and rapamycin (RAPA) alone. CQ was used as a lysosomal inhibitor to block autophagosome degradation, whereas RAPA was used as a positive control for autophagy induction. In the xeno‐MT+CQ group, CQ was administered 2 h after xeno‐MT treatment. RAPA was administered without xeno‐MT. After a total of five treatment cycles, tissues were collected 4 h after the final intervention for protein extraction and western blot analysis. Proteins were extracted from tissue samples using the Protein Extraction Kit (GPP1815, GenePool, China) and denatured at 100°C for 10 min. SDS‐PAGE gels (12% for p62, 15% for LC3B) were prepared and used for electrophoresis at 80 V for the stacking gel and 120 V for the separating gel. Proteins were transferred to a PVDF membrane at 300 mA, with p62 transferred for 2 h and LC3B for 0.5 h. The membrane was blocked in Milk or BSA Blocking Buffer for 1 h. Primary antibodies were diluted in 1% BSA/5% milk: LC3B antibody (ab48394, Abcam) at 1:1000, p62 antibody (39749, CST) at 1:1000, and Actin antibody (ab8226, Abcam) at 1:3000, and incubated overnight at 4°C. After washing, secondary antibodies (goat anti rabbit‐HRP or goat anti‐mouse‐HRP) were diluted 1:5000 and incubated for 50 min at room temperature. The membrane was washed again and developed using ECL. Band intensity was measured using Quantity One v.4.6.2 software.

In addition to the western blot‐based autophagic flux analysis, aged mice were assigned to four groups for functional assessment: control, CQ only, xeno‐MT, and xeno‐MT+CQ. ATP content, ROS levels, and mtDNA copy number were measured in the indicated tissues after 5 times treatment to evaluate whether lysosome‐dependent autophagic flux contributes to the mitochondrial benefits of xeno‐MT.

### 6.14 Western Blot Analysis of Mitophagy and Mitochondrial Dynamic Proteins

In addition to LC3B and p62 for autophagic flux assessment, BNIP3, BCL2, and mitochondrial dynamics proteins were examined as supportive markers for treatment‐associated mitochondrial stress responses and network remodeling. These proteins were not used as direct substitutes for in vivo demonstration of PINK1‐Parkin pathway activation. Protein extraction was performed on tissue samples using the Protein Extraction Kit (GenePool, GPP1815) following the manufacturer's instructions. The protein concentration was adjusted based on loading requirements with water and 5 × SDS‐PAGE Loading Buffer (GenePool, GPP1820), followed by denaturation at 100°C for 10 min. SDS‐PAGE gels were prepared according to the SDS‐PAGE Gel Kit (GPP1816, GenePool, China) protocol, with separating gels of 12% (DRP1, OPA1, MFN1, MFN2, BNIP3, p62), 13% (BCL2), or 15% (FIS1, LC3B, p16, p21) and a 5% stacking gel. Protein samples (35 µg per lane) were loaded, and electrophoresis was conducted at 80 V for stacking and 120 V for separating until the bromophenol blue dye reached the bottom.

For membrane transfer, PVDF membranes (0.22 µm pore size) were pre‐soaked in methanol for 30 s and then transferred into WB Transfer Buffer (GPP1817, GenePool, China). Membranes were assembled with gel and filter paper in a transfer sandwich, ensuring bubble free contact. Wet transfer was performed at a constant current of 300 mA for 2 h. Post‐transfer, membranes were blocked with Milk Blocking Buffer (GPP1819, GenePool,China) or BSA Blocking Buffer (GPP1818, GenePool, China) for 1 h with gentle shaking. Primary antibodies were diluted in 1% BSA or 5% milk and incubated with the membranes overnight at 4°C. The following primary antibodies were used: LC3B (ab48394, Abcam, 1:1000), p62 (39749, CST, 1:1000), BCL2 (ab196495, Abcam, 1:1000), FIS1 (ab189846, Abcam, 1:1000), DRP1 (ab184247, Abcam, 1:1000), OPA1 (ab157457, Abcam, 1:1000), MFN1 (bsm‐60386 M, Bioss, 1:500), MFN2 (ab124773, Abcam, 1:2000), BNIP3 (ab189846, Proteintech, 1:1000), p16 (ab189034, Abcam, 1:500), p21 (28248‐1‐AP, Proteintech, 1:500), GAPDH (ab181602, Abcam, 1:3000) and β‐actin (ab8226, Abcam, 1:3000).

After primary antibody incubation, membranes were washed three times with TBST (GPP1822, GenePool, China, 5 min each) and then incubated with HRP‐conjugated secondary antibodies (anti‐rabbit or anti‐mouse, depending on the primary antibody) diluted 1:5000 in Milk Blocking Buffer for 50 min at room temperature.

Following four washes with TBST (5 min each), membranes were treated with ECL detection reagent (GPP1824, GenePool, China) for 1 min and visualized using film exposure in a darkroom.

### 6.15 Construction of Mitochondrial Heteroplasmic Cell Model

#### 6.15.1 Cell Mitochondria Staining and Enucleation

Yak fibroblasts (Y cells) and the mouse Sertoli cell line TM4 were used to establish the in vitro heteroplasmic model. TM4 cells were selected as a stable and tractable murine recipient cell system that permitted reproducible cell fusion, clonal expansion, and downstream assessment of mitochondrial respiration, ATP, ROS, and mtDNA copy number. In addition, TM4 cells provided a recipient background with relevance to the male reproductive phenotypes examined in vivo. Yak fibroblasts were chosen primarily to maintain donor‐species consistency with the in vivo transplantation experiments and to provide a stable, expandable, and reproducible xenogeneic donor cell source. We note that the purpose of this fusion‐based model was not to identify the donor cell type with the highest mitochondrial abundance, but to establish a controlled and reproducible intracellular xenogeneic mitochondrial‐entry model for mechanistic comparison. Initially, Y and TM4 cells were stained with MitoTracker Deep Red CMXRos and MitoTracker Green FM, respectively, to facilitate subsequent identification and sorting. The mitochondria donor cells, Y cells, underwent denuclearization, following a precise protocol. This included a preparatory step involving a 1:1 mixture of complete medium and percoll, incubated overnight. Subsequently, Cytochalasin B was added to achieve a working concentration of 20 µg/ml. The Y cells were then treated with 0.25% trypsin/EDTA at 37°C for 2 min, followed by a double washing with PBS and centrifugation at 800 g for 5 min. The resulting cell pellet was resuspended in 25 mL of the working solution and incubated for 30 min. This step was critical for the removal of the nucleus, accomplished through ultracentrifugation at 44 000 g at 37°C for 70 min. The layer containing denucleated cells was carefully collected, diluted with ten‐fold the volume of complete medium, and centrifuged at 15 000 g for 5 min. The pellet was then resuspended in 1 mL DMEM for cell counting.

#### 6.15.2 Cell Fusion

TM4 cells, serving as mitochondrial receptors, were then combined with approximately 1 × 10^6 denucleated Y fibroblasts in a 15 mL tube. The cell mixture was centrifuged at 500 g for 5 min. For cell fusion, 100 mL of polyethylene glycol (PEG) was used for resuspension, followed by a 1 min incubation at 37°C. The fusion process was then halted by the addition of 10 mL of complete medium, and the cell mixture was cultured for 24 h.

#### 6.15.3 Fluorescence‐Activated Cell Sorting (FACS)

Prior to Fluorescence‐Activated Cell Sorting (FACS), various samples were prepared, including an unstained Y cell sample, single‐color control samples for compensation (TM4 cells stained with MitoTracker Green FM, and Y cells stained with MitoTracker Deep Red FM), and two‐color stained samples after cell fusion. The post‐fusion cells, displaying dual‐color staining, indicated successful fusion. All samples were collected and processed using a FACSAria Fusion instrument, equipped with four lasers and 15 detectors, and the BD ACDU device for quantitative sorting into a 96‐well plate. FACS parameters were optimized using unstained samples and single staining controls. MitoTracker Green FM positive cells were detected using a blue laser, and MitoTracker Deep Red FM positive cells were detected using a red laser. The selection of dual‐positive cells was based on the simultaneous presence of both MitoTracker signals. These cells, resulting from the fusion of TM4 cells and enucleated Y cells, were isolated into separate wells of a 96‐well plate containing culture medium and were designated as YM cells. Each cell was individually picked using a flow cytometer and seeded into a 96‐well plate. After a 24 h culture period, cells exhibiting both red and green fluorescence under a confocal microscope were confirmed as mitochondrial heteroplasmic cells.

#### 6.15.4 Observation of Cell Fluorescence

The cells were cultured in 96‐well plates for a duration of either 24 or 48 h. Subsequently, cell fluorescence was meticulously observed and captured employing a state‐of‐the‐art super‐resolution laser confocal microscope (Model A1HD25, Nikon, Japan). Perform a secondary selection of cells with both fluorescence signals and continue culturing them. fourth generation of YM cells named YM1 and 11th generation of YM cells named YM2.

### 6.16 OCR Measurement and Cytochrome c Respiration Challenge

The XF96 Extracellular Flux Analyzer (Seahorse XFe96, Agilent, USA) was used to measure bioenergetic function as described [52]. In brief, 2 × 10^4^/well TM4, YM1 or YM2 were plated in XF96 plate and cultured for 24 h. Then, cells were washed with assay medium (Seahorse Medium supplement with 10 mm glucose, 2 mM glutamine and 1 mm pyruvate, pH = 7.4, 37°C) and then placed at 37°C in a CO^2^‐free incubator for 1 h. Oxygen consumption rate (OCR) were obtained with the sequential use of various compounds used to examine the bioenergetic profile.

Mitochondrial stress test was used to estimate mitochondrial function after the sequential use of 2 µm oligomycin, 1 µm FCCP (an uncoupling agent, disrupting the mitochondrial membrane potential) and 0.5 mm rotenone/antimycin A (to inhibit complex I and complex III and to calculate both the mitochondrial and non‐mitochondrial fractions contributing to respiration). Bioenergetic parameters were normalized to 10^4^ cells per well which determined by Cell Proliferation Assay Kit (Thermo Fisher, USA) as a standard. The experiments were performed in three replicates.

The respiratory competence of Fresh‐mito, RT‐2h‐mito, RT‐6h‐mito, RT‐12h‐mito, and Disrupted‐mito was assessed using the extracellular flux analyzer. OCR was recorded sequentially under basal conditions and after the addition of cytochrome c, oligomycin, FCCP, and rotenone/antimycin A. For each assay, an equal amount of mitochondrial preparation normalized by protein concentration was loaded into each well. After baseline respiration had stabilized, cytochrome c was added to assess outer membrane integrity. The final concentrations of cytochrome c, oligomycin, FCCP, and rotenone/antimycin A were the same as those used in the OCR assay setup described above. The cytochrome c response was quantified as the absolute OCR increase after cytochrome c addition.

### 6.17 Comparative Analysis of the Beneficial Effects of Xeno‐MT and Allogenic Mitochondrial Transplantation (Allo‐MT)

Mitochondria were isolated from the skeletal muscle of 3‐month‐old donor mice, resuspended in saline, and intravenously administered to 18‐month‐old recipient mice at a dose of 10 mg per kg of body weight (Allo‐MT). Injections were performed every other day for a total of ten administrations. Age‐matched control mice received an equivalent volume of saline on the same schedule. For the Xeno‐MT group, yak‐derived mitochondria were administered at the same dosage and frequency. All mice were euthanized for analysis 7 days after the final injection.

### 6.18 Mitochondrial Transplantation Using Mitochondria With Impaired Membrane Potential

Mitochondria were isolated from donor cells as described above and immediately resuspended in sterile saline for subsequent experiments. To generate mitochondrial preparations with progressively increased room‐temperature damage, freshly mitochondria preparation were maintained at room temperature for 2, 6, or 12 h, and were designated as RT‐2h‐mito, RT‐6h‐mito, and RT‐12h‐mito, respectively. Freshly mitochondria preparation processed without room‐temperature incubation were designated as Fresh‐mito. To generate a fully disrupted mitochondrial fraction, freshly mitochondria preparation were subjected to three freeze‐thaw cycles followed by sonication, and this preparation was designated as Disrupted‐mito. All mitochondrial preparations were normalized by protein concentration before downstream OCR assays or in vivo administration.

MitoTracker was employed to stain the mitochondria of Y cells. Then mitochondria preparation from Y cells were stranded at room temperature for 2 h (RT‐2h‐mito). Then added to TM4 cells in culture. After 12 h of MT, the plates were washed twice with PBS, and cells were maintained in culture for further analysis. The TM4 received mitochondria named RT‐YM. The room‐temperature‐compromised mitochondria were also transplanted to aged mice by i.v. injection. Following a total of 5 times transplantation, the mice were sacrificed, brain and muscle were selected for further analysis.

All preparations described above are referred to as mitochondria‐enriched preparations, and not as fully purified organelle fractions.

### 6.19 In vivo Validation of Fresh, Room‐temperature‐compromised, and Disrupted Mitochondrial Preparations

To evaluate whether progressively damaged mitochondrial preparations retained in vivo efficacy, aged mice were randomly assigned to six groups: Control, Fresh‐mice, RT‐2h‐mice, RT‐6h‐mice, RT‐12h‐mice, and Disrupted‐mice. Fresh‐mito, RT‐2h‐mito, RT‐6h‐mito, RT‐12h‐mito, and Disrupted‐mito were administered by intravenous injection at the same dose used elsewhere in the study (10 mg/kg body weight). Mice received five administrations at two‐day intervals. Control mice received an equivalent volume of saline on the same schedule. At the same time point after the final injection as described in the original experiment, mice were euthanized and brain and skeletal muscle tissues were collected for biochemical analysis. ATP content, ROS levels, and mtDNA copy number were determined in the indicated tissues using the same procedures described above.

### 6.20 The Effectiveness of Mitochondrial Transplantation using Different Methods and Mitochondria from Various Sources

Mitochondria from Yak cells were stained with MitoTracker. After mitochondrial isolation, the resulting heteroplasmic cells from co‐culture with recipient cells were designated as Co‐culture‐Derived Heteroplasmic Cells (CDHCs‐Y), while those obtained through cell fusion were termed Cytoplast‐Fused Heteroplasmic Cells (CFHCs‐Y). MitoTracker fluorescence images were captured using confocal microscopy, and the fluorescence intensity around each cell nucleus was quantified using Image J software. Mitochondria from HEK293T (CDHCs‐ HEK293T), HepG2 (CDHCs‐ HepG2), and HeLa (CDHCs‐ HeLa) cells were also transplanted into TM4 cells through a co‐culture method.

### 6.21 Relative Quantification of mtDNA Copy Number of Mitochondrial Heteroplasmic Cells

DNA from each TM4, mitochondrial heteroplasmic cells, and mouse tissues was extracted with universal genomic DNA Kit (DN07, Aidlab, China) according to the manufacturer's instructions. To detect specific mtDNA of yak or mice in mitochondrial heteroplasmic cells, the specific primers for yak mtDNA (YND1) and mice mtDNA (MND1) were designed using Primer Premier 5.0. The mice β‐actin gene (MACTB) was used as the internal reference gene, YND1 was used to quantify the yak mtDNA in YM cells, MND1 was used to quantify the mice mtDNA in TM4, mitochondrial heteroplasmic cells, and mouse tissues. qPCR was performed using LightCycler480 fluorescence quantitative PCR instrument (Rotkreuz, Switzerland). The reaction system was 10 µL, including 5 µL of 2 x SYBR Green qPCR Mix (Aidlab, Beijing, China), 3.6 µL of RNase‐free water, 0.2 µL of forward and reverse primers, and 1 µL of DNA. The qPCR program was as follows: 95°C 3 min; 95°C 15s; 60°C 30s, a total of 45 cycles. The relative quantification of mtDNA copy number was calculated by 2^−ΔCT^ method. 3 biological replicates per group and 3 technical replicates per sample were used. Primer sequences are shown in Table S1.

To detect specific mtDNA of yak or mice in mouse tissues, primers named nPCR‐2 (for yak mtDNA) and mt‐mice (for mice mtDNA) were used. The sequences were shown in Table S1.

### 6.22 JC‐10 Fluorescent Assay for Measuring Mitochondrial Membrane Potential

Mitochondria preparation was evaluated using the JC‐10 fluorescent assay for mitochondrial membrane potential (ENZ‐52305, Enzo Life Sciences, USA) according to the manufacturer's instructions. The fluorescent intensities for aggregates (red FL, 590 nm) and monomers (green FL, 520 nm) were measured. Mitochondrial membrane potential (MMP) was determined based on the fluorescence ratio using a fluorescence microplate reader. The percentage of membrane potential was represented as the FL590/FL520 ratio.

### 6.23 Immunofluorescence

Mouse brain and muscle sections, YM1 and RT‐YM cells climbing films were washed with PBS and fixed in 4% paraformaldehyde for preservation. Paraffin sections were deparaffinized by sequentially immersing them in xylene I for 15 min, xylene II for 15 min, absolute ethanol I for 5 min, absolute ethanol II for 5 min, 85% ethanol for 5 min, 75% ethanol for 5 min, and washing with distilled water. Antigen retrieval was performed by placing the sections in a repair box filled with EDTA antigen retrieval buffer (pH 8.0) and heating in a steamer at 95°C for 30 min, preventing excessive evaporation and avoiding drying of the sections. After natural cooling, the slides were washed three times in PBS (pH 7.4) on a decolorizing shaker for 5 min each. To quench autofluorescence, sections were circled with a histochemical pen, incubated with autofluorescence quenching agent for 5 min, and rinsed under running water for 10 min. Sections were then blocked with 5% BSA at room temperature for 30 min. Primary antibody diluted in PBS (LC3B antibody at 1:1000, Abcam/ab48394; PRKN antibody at 1:1000, Abcam/ab15954), and the sections were incubated flat in a humid box at 4°C overnight. After washing three times in PBS, the appropriate secondary antibody was added and incubated in the dark at room temperature for 1 h. Sections were washed three times in PBS and incubated with DAPI staining solution in the dark at room temperature for 10 min. Finally, sections were washed again and mounted with anti‐fluorescence quenching mounting medium.

YM1 or RT‐YM cells were fixed using ice‐cold methanol at 4°C. Permeabilization was achieved with 0.1% Triton X‐100 in PBS, followed by blocking with 5% horse serum for 1 h. Cells were then incubated overnight at 4°C with the LC3B and PRKN antibody. After three PBS washes, cells were incubated with appropriate secondary antibodies at room temperature for 1 h. Following another three PBS washes, nuclear staining was performed using DAPI for 15 min. The Pearson Correlation of different fluorescence was determined by Image J.

### 6.24 Statistical Analysis

Statistical analyses were performed using R packages. For comparisons between two independent groups, data were analyzed using two‐tailed unpaired Student's *t* test when normality assumptions were met; otherwise, the Mann–Whitney *U* test was used. For comparisons involving more than two groups, one‐way ANOVA followed by Tukey's multiple comparisons test was used, or Kruskal–Wallis followed by Dunn's multiple comparisons test when appropriate. For experiments involving two experimental factors, including the age‐by‐treatment design in Figure 3, two‐way ANOVA followed by Sidak's multiple comparisons test was used. For repeated measurements over time, mixed‐effects models or repeated‐measures ANOVA were applied as appropriate.

To address multiple testing across tissues, p values for treatment comparisons within each endpoint family (e.g., ATP, ROS, mtDNA copy number) were additionally adjusted using the Benjamini–Hochberg procedure. For omics analyses (RNA‐seq, WGBS, WGS), false discovery rate (FDR) correction was applied as described in the corresponding subsections. Exact n values are provided in the figure legends. Data are presented as mean ± SEM unless otherwise stated.

## Conflicts of Interest

The authors declare no conflict of interest.

## Supporting information




**Supporting File**: advs75806‐sup‐0001‐SuppMat.docx.

## Data Availability

The data that support the findings of this study are available in the supplementary material of this article.
