# Supplementary material for: Xenogeneic Mitochondrial Transplantation Improves Selected Age‐Associated Phenotypes in Mice
Source: Adv Sci (Weinh). 2026 May 22;13(46):e75806. doi: 10.1002/advs.75806 (PMC13336034; doi:10.1002/advs.75806)
Supplement: Supplementary file 1 [file ADVS-13-e75806-s001.docx]

**Extended Data Figure. 1**

Phenotypic improvements and molecular detection of exogenous mitochondria in progeny. (A) Representative image of an 18-month-old control mouse (left) and an 18-month-old xeno-MT-treated mouse (right). (B) Live birth rate (the number of live-born pups per litter) between xeno-MT-treated and control groups. n = 10 biologically independent samples. (C) Detection of exogenous mtDNA using nest-PCR, 1-11 represent pups’ number, P represents positive control, N represents negative control, M represents marker. (D)The number of positive droplets detected in 11 pups using droplet digital PCR (ddPCR).

**Extended Data Figure. 2**

The number of positive droplets detected in 11 different organs or tissues using droplet digital PCR (ddPCR) in a test mice treated for 1 week.

Extended Data Figure. 3

Effects of mitochondrial transplantation on ATP content, ROS content and mtDNA copy number of mouse tissues. Statistical significance was assessed within each tissue using two-way ANOVA with age and treatment as factors, followed by Sidak’s multiple comparisons test for pre-specified within-age contrasts. Data are expressed as mean ± SEM, *p<0.05.

Extended Data Figure. 4

Effects of mitochondrial transplantation on genome-wide variants. (A) Number of significant variants between A and AC groups. (B) Number of significant variants between AC and YC groups. (C) Number of significant variants between A and YC groups. (D) Significant InDels enriched in GO terms between A and AC groups. (E) Significant InDels enriched in GO terms between AC and YC groups.

Extended Data Figure. 5

The analysis of mitochondrial genome variant counts. (A) InDel counts in muscles between A, AC and YC groups. (B) InDel counts in brains between A, AC and YC groups. (C) SNP counts in muscles between A, AC and YC groups. (D) SNP counts in brains between A, AC and YC groups. (E) Sliding window analysis of the muscle mitochondrial genome SNPs across A, AC and YC groups. (F) Sliding window analysis of the brain mitochondrial genome InDels across A, AC and YC groups.

Extended Data Figure. 6

Insights into the gene expression and methylation induced by mitochondrial transplantation. (A) The number of significant differently expressed genes between A and AC groups. (B) KEGG pathways enriched by significant differently expressed genes in muscle between A and AC groups. (C) KEGG pathways enriched by significant differently expressed genes in brain between A and AC groups. (D) The number of significant differently methylated regions between A and AC groups. (E) KEGG pathways enriched by significant differently methylated genes in muscle between A and AC groups. (F) KEGG pathways enriched by significant differently methylated genes in brain between A and AC groups.

Extended Data Figure. 7

The detection of autophagy protein LC3. (A, B) Immunofluorescence of LC3B in brain and muscle. (C) LC3B particles per cell in brain and muscle. Statistical significance was calculated via two-tailed unpaired Student’s t-test. Data are expressed as mean ± SEM. **p<0.01, ****p<0.0001. Scale bars = 50 µm.

Supplementary Table. 1

18-month-old mice were scored for conspicuous signs of the indicated ageing parameters (0 (none) to 3 (high)).

| Phenotype | A1 | A2 | A3 | A4 | A5 | C1 | C2 | C3 | C4 | C5 |
| --- | --- | --- | --- | --- | --- | --- | --- | --- | --- | --- |
| Alopecia | 1 | 0 | 1 | 1 | 0 | 1 | 1 | 1 | 1 | 0 |
| Greying hair | 0 | 1 | 1 | 0 | 1 | 0 | 1 | 1 | 2 | 2 |
| Kyphosis | 0 | 1 | 1 | 0 | 0 | 1 | 2 | 2 | 0 | 1 |
| Body size reduced | 0 | 0 | 1 | 0 | 1 | 1 | 1 | 1 | 1 | 1 |
| Total | 1 | 2 | 4 | 1 | 2 | 3 | 5 | 5 | 4 | 4 |

Supplementary Table. 2

Primer sequences in this study.

| Primer name | Primer sequences （5’-3’） | Product size （bp） |
| --- | --- | --- |
| ddPCR-*ND1* | F-ATACAACTCCGAAAAGGTCCAA  R-GCAGGTCGTAATGGTTCTTTG | 100bp |
| *ND1*-probe | FAM- CATATGGCCTACTCC -MGB |  |
| qPCR-*ND1* | F- GCCAGCCTGACCCATAGCCATAAT  R- GCCGGCTGCGTATTCTACGTTA | 126bp |
| qPCR-*GAPDH* | F- TGGTGAAGCAGGCATCTGAG  R- GTTGCTGTTGAAGTCGCAGG | 85bp |
| nPCR-1 | F- ATCAAAGAACCATTACGACCTG  R- ATAGCGGAATCGAGGATAGG | 678bp |
| nPCR-2 | F- TAGCCTTAACCATGTGAATT  R- GGGATGTCCCTAGAAATAGA | 498bp |
| Y*ND1* | F- AGCCGTAGCATTCCTCAC  R- ACGACATTTGGACCTTTT | 75bp |
|  |  |  |
| Y*ACTB* | F- TGCGGCATTCACGAAACT  R- CCGTGTTGGCGTAGAGGT | 79bp |
|  |  |  |
| M*ND1* | F- TTGGTCCATACGGCATTT  R- GAGTGATAGGGTAGGTGC | 119bp |
|  |  |  |
| M*ACTB* | F-GCCTTCCTTCTTGGGTAT  R-GTCTTTACGGATGTCAACG | 88bp |
|  |  |  |
| mt-mice | F- TTCCACCAACCAGCATTCCA | 682bp |
|  | R- TCCAGAGACTTGGGGATCTAACT |  |

Supplementary Table. 3

The primers sequence of bisulfite sequencing and PCR procedures.

| Primers | Sequence | | Length | |
| --- | --- | --- | --- | --- |
| F | TATTAATTTGTTAATGTTTATTGCGTAA | | 442bp | |
| R | CGAATCTAATCAACCCATAACCA |  | |  |
| Procedure | Temperature |  | | Time |
| 1. Denaturation | 95 |  | | 3 min |
| 2. Denatured | 94 |  | | 30 sec |
| 3. Annealing | 55 |  | | 30 sec |
| 4. Extension | 72 |  | | 45sec |
| 5. Cycles 2-4 | 35 cycles |  | |  |
| 6. Repair Extension | 72 |  | | 5 min |

**Supplementary Table. 4**

The expression levels of highlighted genes in muscle of aged and control mice.

| Gene Symbol | Amuscle Average TPM | ACmuscle Average TPM | Qvalue (Amuscle / ACmuscle) |
| --- | --- | --- | --- |
| 'Adam8' | 0.643333 | 3.04 | 4.58E-33 |
| 'Capn6' | 3.523333 | 1.29 | 2.51E-29 |
| 'Cd1d1' | 5.096667 | 1.503333 | 2.95E-27 |
| 'Cxcr4' | 3.05 | 6.783333 | 8.30E-15 |
| 'Dnm1' | 5.16 | 2.07 | 7.79E-41 |
| 'Gpc3' | 8.703333 | 4.373333 | 7.37E-25 |
| 'Hck' | 0.84 | 2.76 | 3.85E-13 |
| 'Il1b' | 0.76 | 4.456667 | 7.95E-22 |
| 'Napsa' | 0.626667 | 3.22 | 1.20E-15 |
| 'Lrp1' | 8.843333 | 4.33 | 1.54E-201 |
| 'Mmp13' | 0.063333 | 3.593333 | 1.72E-67 |
| 'Mpo' | 0.153333 | 5.823333 | 7.03E-102 |
| 'Mrc2' | 3.273333 | 1.62 | 1.06E-27 |
| 'Prtn3' | 0.44 | 7.273333 | 3.24E-34 |
| 'Slc2a6' | 0.22 | 0.733333 | 1.09E-04 |
| 'Trpm2' | 0.083333 | 1.473333 | 1.18E-41 |
| 'Mefv' | 0.05 | 0.54 | 2.84E-10 |
| 'Acpp' | 0.07 | 0.356667 | 9.37E-06 |
| 'Entpd4' | 2.53 | 1.106667 | 1.37E-14 |
| 'Ifitm1' | 3.406667 | 11.83 | 1.71E-12 |
| 'Unc13d' | 0.203333 | 0.733333 | 6.19E-09 |

**Supplementary Table. 5**

The expression levels of highlighted genes in brain of aged and control mice.

| Gene Symbol | Abrain Average TPM | ACbrain Average TPM | Qvalue (Abrain / ACbrain) |
| --- | --- | --- | --- |
| 'Agt' | 21.6 | 51.21333 | 2.66E-92 |
| 'Atp2a1' | 0.26 | 1.126667 | 6.54E-09 |
| 'Dlk1' | 0.86 | 2.7 | 3.60E-23 |
| 'Slc17a6' | 23.59 | 46.99667 | 2.57E-137 |
| 'Ido1' | 4.76 | 1.963333 | 1.53E-06 |
| 'Dnm1' | 221.98 | 178.2666 | 2.18E-04 |
| 'Prkcd' | 35.35667 | 86.99667 | 1.22E-265 |
| 'Ret' | 1.576667 | 3.14 | 4.59E-18 |
| 'Magel2' | 0.346667 | 0.84 | 7.95E-05 |
| 'Baiap3' | 3.043333 | 7.113333 | 1.81E-36 |
| 'Rab37' | 6.623333 | 14.99 | 1.53E-29 |
| 'Ifi27l2a' | 22.68 | 10.30667 | 9.94E-04 |

Supplementary Data. 1

Uncropped immunoblots from Figure 5.

Supplementary Data. 2

Uncropped immunoblots from Figure 6.

Supplementary Data. 3

Uncropped immunoblots from Figure 8.

Supplementary Data. 4

Uncropped immunoblots from Figure 9.
